# Inferring disease progression stages in single-cell transcriptomics using a weakly supervised deep learning approach

**DOI:** 10.1101/gr.278812.123

**Published:** 2025-01

**Authors:** Fabien Wehbe, Levi Adams, Jordan Babadoudou, Samantha Yuen, Yoon-Seong Kim, Yoshiaki Tanaka

**Affiliations:** 1 Maisonneuve-Rosemont Hospital Research Center (CRHMR), Department of Medicine, University of Montreal, Quebec H1T 2M4, Canada;; 2 RWJMS Institute for Neurological Therapeutics, Rutgers–Robert Wood Johnson Medical School, Piscataway, New Jersey 08854, USA;; 3 Department of Biology, Bates College, Lewiston, Maine 04240, USA

## Abstract

Application of single-cell/nucleus genomic sequencing to patient-derived tissues offers potential solutions to delineate disease mechanisms in humans. However, individual cells in patient-derived tissues are in different pathological stages, and hence, such cellular variability impedes subsequent differential gene expression analyses. To overcome such a heterogeneity issue, we present a novel deep learning approach, scIDST, that infers disease progression levels of individual cells with weak supervision framework. The disease progression–inferred cells display significant differential expression of disease-relevant genes, which cannot be detected by comparative analysis between patients and healthy donors. In addition, we demonstrate that pretrained models by scIDST are applicable to multiple independent data resources and are advantageous to infer cells related to certain disease risks and comorbidities. Taken together, scIDST offers a new strategy of single-cell sequencing analysis to identify bona fide disease-associated molecular features.

Over the past few years, single-cell technologies have rapidly advanced and been applied to patient-derived tissues to better understand and counter a variety of diseases (BRAIN Initiative Cell Census Network (BICCN) 2021). Comparative analysis with healthy donors’ data is widely implemented to identify potential disease-associated genes (Velmeshev et al. 2019; Adams et al. 2024). However, the patient-derived biospecimen is composed of a mixture of cells in various pathological stages and even contains healthy cells. Such heterogeneity obscures differential expression between patient and healthy donors and hinders identification of bona fide disease-associated gene expression patterns (Trapnell 2015).

Deep learning is a type of artificial intelligence (AI) method that automatically recognizes feature trends and patterns from data sets and solves a complex classification and regression problem (LeCun et al. 2015). Deep learning has been widely implemented in various single-cell data analyses, including data imputation (Arisdakessian et al. 2019), doublet identification (Bernstein et al. 2020), dimensionality reduction (Deng et al. 2019), batch effect corrections (Xu et al. 2022), and cell type annotations (Brbić et al. 2020; Lotfollahi et al. 2022). Despite these broad applications, there is a limited application of deep learning to the inference of disease progression of individual cells in the patient-derived data (Brendel et al. 2022). One of major challenges may be difficulty to train the model from the binary diagnostic information (e.g., patient = 1 or healthy donor = 0) and regress it to continuous disease progression levels (from early to progressive stage). To overcome such issues, we propose a novel approach, single-cell identification of disease progression stage (scIDST), that infers the disease progression levels of individual cells in single-cell transcriptome profiles with weakly supervised deep learning. The weak supervision models utilize labeling functions that are automatically generated from a small subset of labeled data sets and give weak labels on large unclear data sets (Varma and Ré 2018). These weak labels are subsequently used for training of a machine learning classifier. Consequently, the classifier model is trained by a weaker form of supervision than the conventional supervised learning and is less vulnerable to overfitting to inaccurate original binary labels. Here, using these weakly supervised deep learning models, we aim to (1) functionally segregate cells with aberrant expression of the disease-relevant genes, (2) infer diseased cells across different data sources, and (3) infer cells associated with comorbidity or certain symptoms of diseases.

## Results

### Limitation of clustering-based approach to classify cells by disease status

Conventionally, the cellular heterogeneity across the patient-derived single-cell data has been dissected by clustering-based approaches (Hafemeister and Satija 2019; Smajić et al. 2022; Smith et al. 2022). To identify disease-specific cellular states, we performed the clustering analyses (e.g., graph-based clustering by Seurat) in single-cell RNA-seq of the midbrain from Parkinson's disease (PD) patients and healthy young and aged donors (Adams et al. 2024). However, uniform manifold approximation and projection (UMAP) separated individual cells by cell types, irrespective of disease status (Supplemental Fig. S1A). The clustering further divided seven cell types into 23 distinct clusters (Supplemental Fig. S1B) but failed to identify clusters that are unique or predominant to the PD patients (Supplemental Fig. S1C). In addition, the number of differentially expressed genes was inversely correlated with the size of clusters (Supplemental Fig. S1D) and cell types (Supplemental Fig. S1E), suggesting the limitations of the clustering-based methods in the context of the classification by disease status (Trapnell 2015). This statistical issue gives rise to limited differential expression of relevant PD-related genes, such as *SNCA* and *MAPT* (Supplemental Fig. S1F; Klein and Westenberger 2012). Importantly, in the PD brain, the expression of these genes shows cellular variability and is correlated with loss of neuronal connection strength (Rittman et al. 2016). Therefore, to robustly uncover disease-associated molecular elements from single-cell data, it is critical to functionally segregate cells based on disease progression levels.

### Impediment of supervised deep learning in single-cell analysis

**Figure 1.**
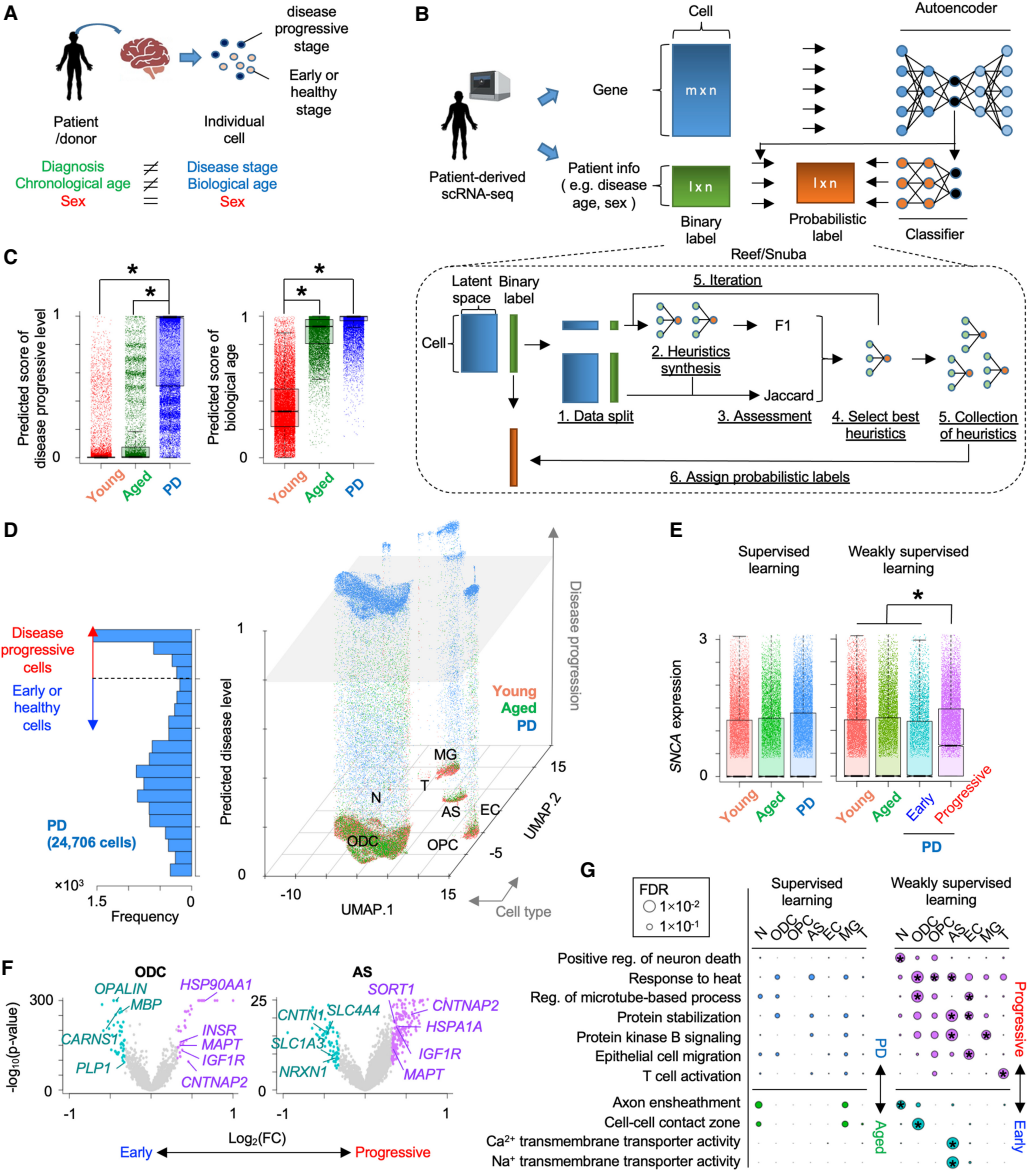
Inference of Parkinson's disease (PD) progression levels of individual cells from patient-derived scRNA-seq. (*A*) Difference between patient information and status of individual cells in patient-derived tissue. (*B*) Schematic of weakly supervised deep learning model to infer disease-progressed cells. (*C*) Boxplots showing predicted score of disease progressive level (*left*) and biological age (*right*). (*) *P* < 0.05 by two-sided *t*-test. (*D*) 3D plot showing disease-progressed cells in each cell type. The *x*- and *y*-axes represent the first and second dimensions of UMAP. The *z*-axis is the predicted disease progression level. Histogram of predicted disease level in PD patient–derived cells is shown in *left* panel. The dashed line and gray plane represent threshold of disease progression and early-staged/healthy cells. (*E*) Comparison of synuclein alpha (*SNCA*) expression across groups that are segregated by supervised (*left*) or weakly supervised (*right*) deep learning. (*F*) Volcano plots showing differential expression between PD progressive and early-staged/healthy cells. (*G*) Circle plot showing overrepresented GO terms between PD progressive and early-staged/healthy cells (*right*) but not between PD and aged brain (*left*). Circle size represents –log_10_(FDR). Statistical significance (FDR < 0.05) is shown by an asterisk. (N) Neuron, (ODC) oligodendrocyte, (OPC) oligodendrocyte precursor cell, (AS) astrocyte, (EC) endothelial cell, (MG) microglia, and (T) T cell.

To segregate the diseased cells from the early-staged/healthy ones in PD brains, we first applied the deep learning model to the PD single-cell transcriptome data using patient information (disease diagnosis, age, and sex) as binary training data label (0 or 1; see Methods) (Supplemental Fig. S1G). Although the deep learning model separated cells of PD patients from those of healthy donors and separated cells of aged donors from those of young donors with >93% accuracy (Supplemental Fig. S1H), the prediction performance for disease or age was significantly lower than that for sex (Supplemental Fig. S1I). Importantly, individual cells in patient-derived tissue are in various disease progression stages and biological ages (Auerbach et al. 2021; Zhang et al. 2021), whereas sex is uniform among cells in one patient (Fig. 1A). Therefore, the supervised binary classification model is not appropriate to infer such heterogeneous features, and it remains challenging to maximize the performance of deep learning in the context of the single-cell data analysis.

### Weak supervision improves estimation of disease progression levels

To overcome the conflicts between the machine learning ability and single-cell analyses, we redesigned our deep learning model by employing weakly supervised learning (Fig. 1B; Ratner et al. 2020). The weak supervision first estimates the accuracy of imperfect data source and converts the binary label (0 or 1) into probabilistic label (0∼1) (for details, see Methods). The probabilistic labels are calculated by the Reef/Snuba system, which automatically generates the labeling functions from a small portion of the data sets and iteratively adjust parameters by pruning low-confidence labeling functions (Varma and Ré 2018). The classifier model is in turn trained from the probabilistic labels and finally provides scores representing if a cell is likely to be diseased or aged. Unlike the supervised learning approach (Supplemental Fig. S1H), the weakly supervised deep learning model outputted more broad prediction scores (Fig. 1C; Supplemental Fig. S2A). Particularly, the predicted disease score displayed bimodal distribution in PD patient–derived cells and separated cells into two groups (Fig. 1D): disease-progressed and early-staged/healthy cells. Importantly, vast majority of cells with a higher disease score belonged to PD, and a few of them were also observed in healthy aged donors (Supplemental Fig. S2B). The high-scoring cells displayed significantly elevated expression of synuclein alpha (*SNCA*), a common pathological hallmark of PD (Fig. 1E; Supplemental Fig. S2C; Klein and Westenberger 2012). Furthermore, other PD-relevant (*MAPT*, *IGF1R*, *INSR*, *HSPA1A*, *HSP90AA1*, *CNTNAP2*, and *CNTN1*) (Uryu et al. 2006; Klein and Westenberger 2012; Infante et al. 2015; Castilla-Cortazár et al. 2020; Chatterjee et al. 2020), myelination-related (*MBP*, *PLP1*, and *OPALIN*), and astrocytic transporter (*SLC1A3* [also known as *GLAST*] and *SLC4A4*) genes were also differentially expressed between disease-progressed and early-staged/healthy oligodendrocytes or astrocytes from PD patients (Fig. 1F). In the PD brain, heat shock chaperone levels are increased in response to pathological aggregates of SNCA protein (Uryu et al. 2006). Gene Ontology (GO) analysis revealed that heat shock–related genes were significantly elevated in PD progressive glial cells, whereas no significant difference was observed, when globally comparing cells between PD patients and aged-matched donors (Fig. 1G). Genes related to other PD hallmarks, such as neuronal death (Klein and Westenberger 2012), *N*-methyl-D-aspartate (NMDA) activation (Ahmed et al. 2011), and T cell activation (Lindestam Arlehamn et al. 2020), were also increased in the disease-progressed neurons and T cells (Fig. 1G; Supplemental Fig. S2D,E). In addition, the weakly supervised deep learning estimated the biological age of individual cells. Importantly, the inferred biological age was positively correlated with *FKBP5* gene expression, which is known to be elevated by aging (Supplemental Fig. S2F; Zannas et al. 2019). We also note that scIDST displayed superior or comparable performance to the existing tools in the inference of disease progression and biological aging (see Supplemental Note). Overall, the weakly supervised model offers a robust platform to dissect patient-derived single-cell data and provides new insights into human disease mechanisms.

### scIDST can infer disease progression levels across multiple data sets

**Figure 2.**
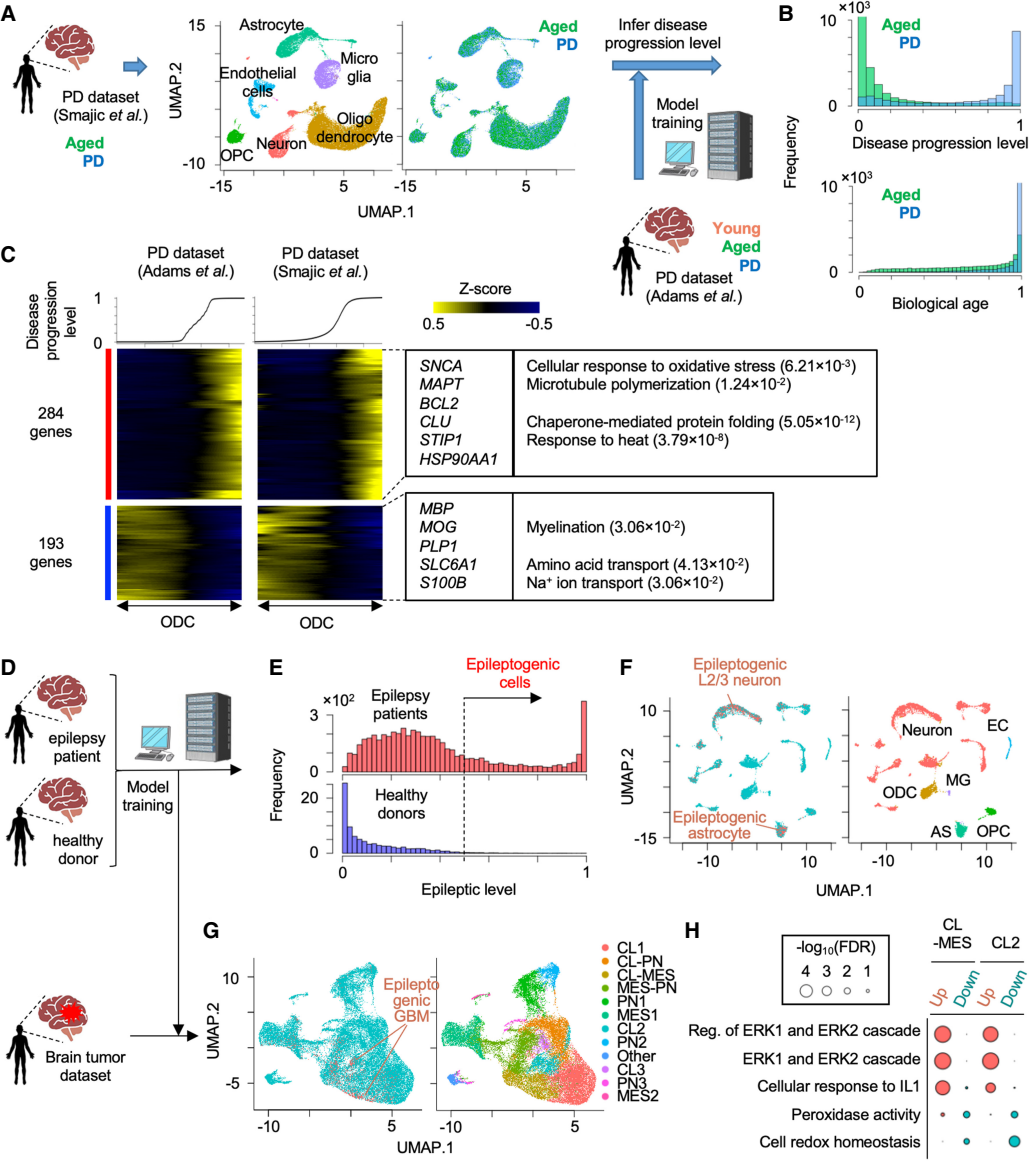
Application of pretrained weakly supervised models to other independent single-cell data sets. (*A*) Strategy to estimate PD progressive level by the pretrained model in another independent single-cell transcriptome data set. (*B*) Histogram of predicted score of disease progression level and biological age. (*C*) Heatmap showing genes whose expression is highly correlated with an inferred disease progression score (genes with Pearson's correlation > 0.1 in both data set A and B are used). Oligodendrocyte cells are sorted by the disease progression score. Heatmap color represents z-score-normalized gene expression. Representative genes and GO terms are shown in *right* panel. Statistical significance of GO terms is shown by the false-discovery rate (FDR). (*D*) Strategy to infer epileptogenic cells in GBM tissues using weakly supervised deep learning model. (*E*) Histogram showing the inferred epileptic levels of individual cells in epilepsy patients and healthy donors. Dashed line represents threshold to define epileptogenic cells. (*F*) UMAP plot of single cells colored by epileptogenic (red) and nonepileptogenic cells (blue; *left*) and cell types (*right*) in scRNA-seq of epilepsy patients and healthy donors. (*G*) UMAP plot of single cells colored by epileptogenic (red) and nonepileptogenic cells (blue; *left*) and clusters (*right*) in scRNA-seq of GBM patients. (*H*) Representative GO terms of differentially expressed genes between epileptogenic and nonepileptogenic cells in CL-MES and CL2 cluster. (CL) Classical, (MES) mesenchymal, and (PN) proneural.

Given the potential of the weak supervision in the disease progression prediction, we tested whether our model is applicable across multiple independent single-cell RNA sequencing (scRNA-seq) data sets. Using the pretrained model of the PD scRNA-seq data set (midbrains of young and aged healthy donors and PD patients) (as used in Fig. 1; Adams et al. 2024), we inferred disease progression levels of individual cells in another independent PD scRNA-seq data set (midbrains of aged healthy donors and PD patients) (Fig. 2A; Smajić et al. 2022). Similarly, the predicted disease progressive scores are broad but significantly higher in PD patients than in aged donors (Fig. 2B). In addition, vast majority of cells in both aged donors and PD patients displayed high scores of biological ages. Our model identified a substantial amount of disease-progressed neurons in the Adams et al. data sets (Supplemental Fig. S2A) but classified vast majority of neurons in the Smajić et al. data sets as early/healthy cells (Supplemental Fig. S3A). The major difference between two data sets was a unique neuronal cluster that was characterized by high expression of *CADPS2* and was not detected in the data set of Adams et al. (Smajić et al. 2022; Adams et al. 2024). Although several studies demonstrated that *CADPS2* expression was aberrantly expressed in PD (Reinhardt et al. 2013), *CADPS2* transcriptional activity was also inversely regulated by *SNCA* (Obergasteiger et al. 2017). Consequently, we found that the Smajić et al. data sets contained a small amount of *SNCA*^high^ neurons, as well as a substantial amount of *CADPS2*^high^ neurons that seems to be intermediate disease state (Supplemental Fig. S3B).

To validate the reliability of the inferred disease score, we identified 284 and 193 genes whose expression in oligodendrocytes were positively and negatively correlated with the disease progression score in both PD data sets, respectively (Fig. 2C). We observed a significant reduction in myelination-related genes (e.g., *MBP*, *MOG*, *PLP1*), hence confirming the pathological feature of neurodegenerative diseases (Ettle et al. 2016). Furthermore, expression of PD-associated (e.g., *SNCA*, *MAPT*) (Klein and Westenberger 2012) and other neurodegenerative disease–associated genes (e.g., *BCL2, CLU*) (Satou et al. 1995; Killick et al. 2014) were significantly elevated with the inferred disease progression but did not display a significant difference by global comparison between PD patients and aged healthy donors (Supplemental Fig. S3C).

In addition to the data set from the same brain region, we also tested whether our model can be used in scRNA-seq data from different brain regions. Using the same pretrained model (Adams et al. 2024), we inferred the disease progression levels and the biological ages in the data set of putamen from aged healthy donors and PD patients (Supplemental Fig. S3D; Xu et al. 2023). As observed in the midbrain data set, the biological ages were similar between PD patients and age-matched healthy donors, whereas the disease progression levels were significantly higher in PD patients (*P* < 2.2 × 10^−16^ by two-sided *t*-test) (Supplemental Fig. S3E). Significant upregulation of PD-related genes and downregulation of myelination-associated genes were also detected along with the inferred disease progression (Supplemental Fig. S3F). Overall, our model did not overfit to a specific single-cell transcriptome data set and can infer the disease progression levels across multiple single-cell data sets.

To expand the practical utility of scIDST, we also tested whether our model is capable of identifying cells associated with certain symptoms and comorbidities using scRNA-seq data sets of different diseases. For example, epileptic seizure is one of the most common symptoms in glioblastoma multiforme (GBM) patients (van Breemen et al. 2007). The vulnerability to seizure is positively correlated with a *EGFR* level that is frequently overexpressed or amplified in GBM patients (Yang et al. 2014). To test predictive performance of the comorbid epilepsy-related cells in GBM tissues, we first trained the weakly supervised deep learning model with scRNA-seq of epilepsy patients and healthy donors (Fig. 2D; Velmeshev et al. 2019). The model clearly separated epileptogenic cells that were enriched in cortical layer 2 and 3 (L2/3) excitatory neurons and astrocytes that are major vulnerable cell types to epilepsy (Fig. 2E,F; Supplemental Fig. S3G,H; Pfisterer et al. 2020; Vezzani et al. 2022). We confirmed that these segregated cells were characterized by aberrant upregulation of genes related to voltage-gated calcium ion channels, glutamatergic synaptic organization, and action potential, which are all hallmarks of neuronal hyperexcitability in epilepsy patients (Supplemental Fig. S3I; Velmeshev et al. 2019). Subsequently, we inferred epileptogenic cells in scRNA-seq of GBM patients using the model trained by the epilepsy data sets (Fig. 2D; Bhaduri et al. 2020). The pretrained model identified the potential epileptogenic GBM cells predominantly in two clusters (CL-MES and CL2) that were characterized by a classical GBM molecular subtype (Fig. 2G; Wang et al. 2017). Differential expression analysis showed that the epileptogenic GBM cells aberrantly elevated ERK1/2 signaling genes that trigger synaptic hyperexcitation in the host neural network, which in turn leads to seizure (Fig. 2H; Nateri et al. 2007). Importantly, *EGFR* is a main upstream regulator of ERK1/2 pathway, and its expression is often elevated in the classical GBM subtype (Verhaak et al. 2010). Accordingly, the inferred epileptogenic GBM cells are predominantly derived from patients with *EGFR* amplification and display significant elevation of *EGFR* gene expression (Supplemental Fig. S3J,K). Taken together, these results suggest that our weakly supervised deep learning model has a great potential to infer cellular states associated with certain comorbidity risks and support the investigation of relationships across different diseases.

### Assessing the inferred disease progression with other pathological hallmarks

Clinically, disease progression has been often diagnosed by presence and spread of pathological biomarkers. Alzheimer's disease (AD) is another progressive neurodegenerative disorder and is characterized by amyloid beta plaques (Aβ) and neurofibrillary tangles that are primarily composed of hyperphosphorylated Tau (pTau) protein (Weingarten et al. 1975). Braak staging is one of sensitive and reliable measurements to assess the cognitive and psychological status of AD patients by quantifying topographical distribution of the neurofibrillary tangles in the brain (Braak and Braak 1991). To evaluate the reliability of scIDST-based disease progression prediction, we analyzed the consistency between the inferred disease progression levels and these clinically used biomarkers.

**Figure 3.**
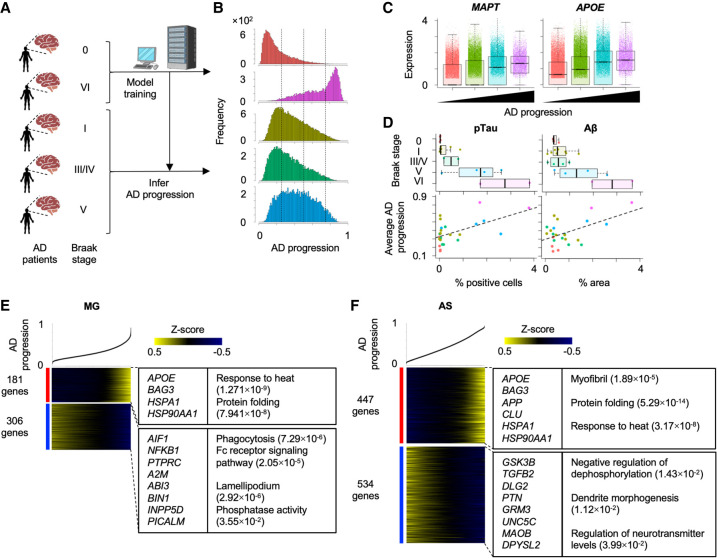
Inference of Alzheimer's disease (AD) progression across different Braak stages. (*A*) Strategy to estimate AD progressive levels in scRNA-seq of patient-derived samples in different Braak stages. The model was trained by Braak stages VI and 0 and estimated disease progression levels in other Braak stages. (*B*) Histograms of AD progressive levels in each Braak stage. Dashed lines are cutoff values to separate cells into four groups. (*C*) Differential expression of *MAPT* and *APOE* across four disease progression groups. (*D*) Comparison of average AD disease progressive levels in each patient and percentages of the pathological aggregates in the brain section. The pathological aggregate levels in each Braak stage are also shown in *top* panel. (*E*,*F*) Heatmap showing genes whose expression is highly correlated with inferred disease progression score (genes with Pearson's correlation > 0.1) in microglia (*E*) and astrocytes (*F*). Cells are sorted by the disease progression score. Heatmap color represents *z*-score-normalized gene expression. Representative genes and GO terms are shown in *right* panel. The statistical significance of GO terms is shown by the FDR.

We trained the weakly supervised deep learning model with scRNA-seq of nondiseased donors and AD patients with the highest Braak stage (VI) (Fig. 3A; Smith et al. 2022). Subsequently, the trained model was used to infer the progressive levels of AD patients with the intermediate Braak stages (I, III/IV, and V). Importantly, the inferred AD progressive levels were significantly correlated with Braak stage of AD patients (Pearson's correlation = 0.481, *P* < 2.2 × 10^−16^) (Fig. 3B). AD-relevant genes (*MAPT* and *APOE*) were significantly elevated with the inferred AD progression (Fig. 3C). In addition, average AD progressive levels in each patient were significantly correlated with quantification values of immunostaining for pTau and Aβ (Pearson's correlation = 0.566 [*P* = 3.93 × 10^−3^] and 0.533 [*P* = 7.24 × 10^−3^] respectively) (Fig. 3D). These results indicate that the inferred disease progression by scIDST is strongly consistent with other pathological quantification methods.

AD often coincides with chronic inflammation that increases neuronal damage and is instigated by aberrant activation of microglia and astrocytes. To dissect the gene expression changes along AD disease progression, we identified 181 and 306 genes in microglia and 447 and 534 genes in astrocytes, whose expression were positively and negatively correlated with the inferred AD progression levels, respectively (Fig. 3E,F). Both cell types displayed significant elevation of heat shock–related genes, which is a major characteristic of neurodegenerative diseases (Uryu et al. 2006). In addition, microglia significantly reduced gene expression related to phagocytosis, which is essential for clearance of pTau and Aβ. In contrast, astrocytes upregulated genes related to Aβ toxicity (*APP* and *CLU*) and downregulated genes related to negative regulation of dephosphorylation (*GSK3B*, *TGFB2*, *DLG2*, and *PTN*) and other AD-related genes (*GRM3*, *UNC5C*, *MAOB*, and *DPYSL2*). These differential expression patterns are consistent with accumulation of pTau and Aβ in AD patients’ brains. Overall, scIDST successfully inferred AD progressive levels of individual cells, which were consistent with the pathological measurements of AD.

### scIDST also can infer cellular response to small molecules

**Figure 4.**
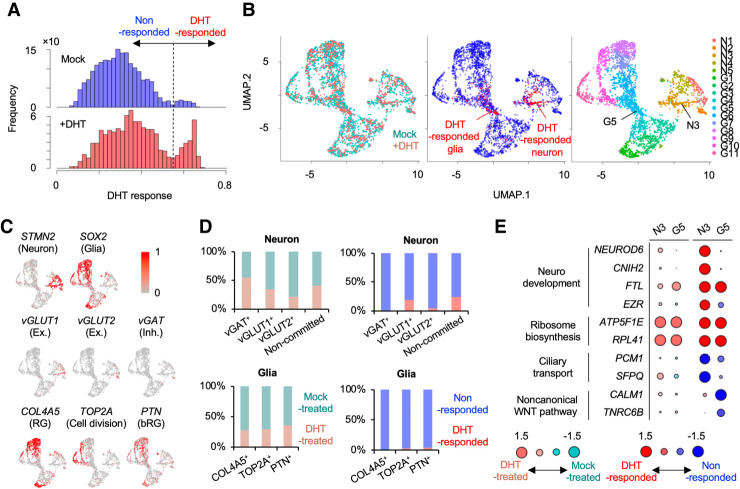
Inference of response to DHT treatment in brain organoids by weakly supervised deep learning. (*A*) Histograms of DHT response score in DHT- and mock-treated organoids. Dashed line is a threshold to define DHT-responsive cells (DHT response score > 0.55). (*B*) UMAP plot of single cells colored by organoid type (*left*), DHT response (*middle*), and cluster (*right*): (N) neuron, (G) glia. (*C*) UMAP plot showing representative gene expression for each cell type. (*D*) Ratio of cells from DHT-treated and mock-treated organoids (*left*) and DHT- and nonresponded cells (*right*) in each cell type. (*E*) Representative differentially expressed genes in N3 and G5 cluster between DHT- and mock-treated cells (*left*) and between DHT- and nonresponded cells (*right*). Circle size represent log2(ratio).

**Figure 5.**
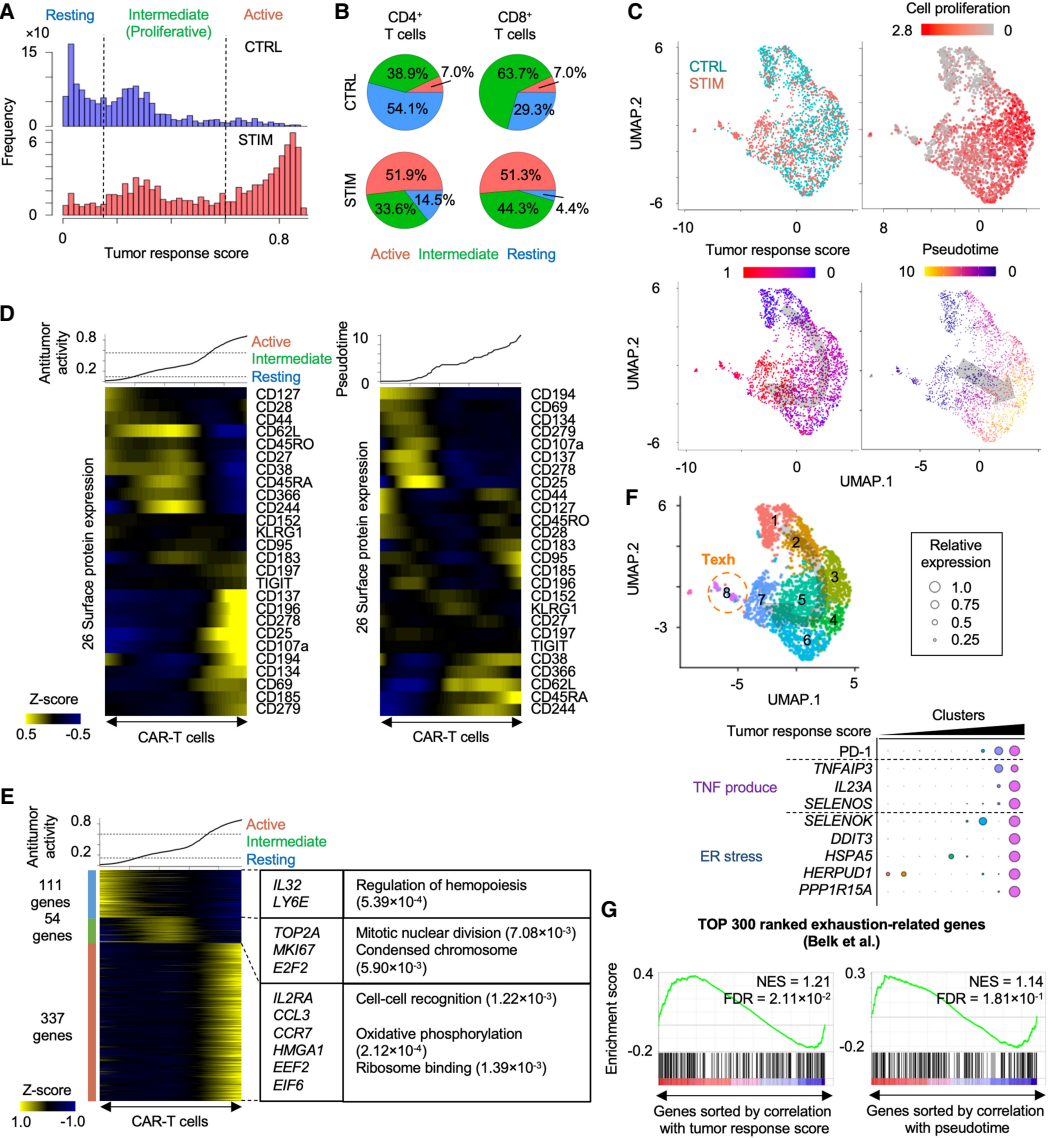
Inference of activation of CAR-T cells by neuroblastoma. (*A*) Histograms showing inferred antitumor activity in neuroblastoma (STIM) and nonstimulated (CTRL) CAR-T cells. (*B*) Pie chart of the ratio of three groups in CD4^+^ and CD8^+^ T cells with and without neuroblastoma stimulation. (*C*) UMAP plots of individual CAR-T cells colored by the stimulation conditions (*top left*), cell proliferation–related gene expression (*top right*), the inferred antitumor activity (*bottom left*), and pseudotime (*bottom right*). (*D*) Heatmap showing 26 surface protein expression dynamics across CAR-T cells. CAR-T cells were sorted by the inferred antitumor score (*left*) or pseudotime (*right*). Heatmap color represents *z*-score-normalized protein expression. (*E*) Heatmap showing differentially expressed genes across three groups. CAR-T cells were sorted by the inferred antitumor score. Representative genes and significant GO terms were shown in *right* panel. (*F*) UMAP plots of individual CAR-T cells colored by clusters (*top*). Representative differentially expressed surface proteins and genes are shown (*bottom*). The cluster number is ordered along the inferred tumor response score. (*G*) Gene Set Enrichment Analysis of T cell exhaustion gene signatures. Genes are sorted by Pearson correlation coefficient between their expression and antitumor score (*left*) or pseudotime (*right*). Normalized enrichment score (NES) and FDR are also shown.

Given the promising performance of scIDST in the inference of disease progression, we next address whether scIDST can infer other heterogeneous phenotypes, such as drug response (Fig. 4) and cellular response to pathogens (Fig. 5). Sexual dimorphism in brain anatomy and network connectivity emerges at developing fetal stage and is expanded with age. An excellent study by Kelava and colleagues (2022) recently demonstrated that androgens specifically accelerate excitatory neuronal potential by treating an androgen steroid hormone, dihydrotestosterone (DHT), to brain organoids. They also performed scRNA-seq to DHT- and mock-treated organoids but identified only a small number of differentially expressed genes with comparing cells between DHT- and mock-treated organoids. Furthermore, most of the differentially expressed genes are related to ribosome biogenesis but are not associated with neuronal excitation or brain development.

Here, we hypothesize that only a portion of cells in the brain organoids responded to DHT, and thereby, differential expression was obscured by direct comparison between DHT- and mock-treated organoids. To infer DHT response level in individual cells, we performed the weakly supervised deep learning to scRNA-seq of DHT- and mock-treated organoids. We observed that a substantial number of cells in DHT-treated organoids displayed high response score (17.6% with >0.55), in which cells with high score are limited in mock-treated organoids (3.99%) (Fig. 4A). These DHT-responded cells were observed in specific neuronal and glial clusters (N3 and G5) (Fig. 4B,C). In neurons, DHT-responded cells were only detected in excitatory and noncommitted neurons (Fig. 4D). This is consistent with the observation of Kelava et al. (2022), in which inhibitory neurons were not responsive to DHT. In addition, as Kelava et al. detected increased glial proliferation and basal progenitors in DHT-treated organoids, DHT-responded cells were detected in *TOP2A*^*+*^-proliferating cells and *PTN*^*+*^ basal radial glia. Importantly, these cell type–specific responses to DHT were not detectable by simple cell composition analysis between DHT- and mock-treated organoids (Fig. 4D, left).

Differential expression analysis between DHT- and nonresponded cells identified a higher number of up- and downregulated genes (331 and 51 genes in N3 cluster, 212 and 129 genes in G5 cluster, respectively) than global comparison between DHT- and mock-treated cells (131 and 14 genes in N3 cluster, 136 and 52 genes in G5 cluster, respectively). We detected significant differential expression of neurodevelopmental genes in DHT-responded neurons (Fig. 4E). In particular, genes associated with male-biased neurological diseases (e.g., *NEUROD6* for autism, *CNIH2* for schizophrenia) (Baxter et al. 2012; Drummond et al. 2012) were differentially expressed between DHT- and nonresponded neurons but not between DHT and mock-treated neurons. Overall, these results indicate that scIDST is a promising tool to identify bona fide differential expression in scRNA-seq analysis.

### scIDST also can infer cellular response to pathogens

Chimeric antigen receptor (CAR)-T cell is genetically engineered immune cell to target specific antigens and a promising immunotherapy for cancer treatment. Clinically, the efficacy and outcome of CAR-T cell therapy are positively associated with expansion and persistence of CAR-T cells (Gumber and Wang 2022). The tumor-stimulated CAR-T cells first enter the proliferative phase and subsequently drive their effector cytotoxic activity (Li et al. 2021). However, prolonged antigen stimulation exhausts CAR-T cells and leads to loss of their effector function and proliferative capacity (Gumber and Wang 2022). Therefore, to overcome the limitation of current CAR-T cell therapy, uncovering of the molecular dynamics of CAR-T cell response is important. Here, we trained the weakly supervised deep learning model with cellular indexing of transcriptomes and epitomes by sequencing (CITE-seq) of CAR-T cells stimulated with or without neuroblastoma cells, and inferred tumor response level of individual cells (Tian et al. 2022). The inferred tumor response score displayed trimodal distribution and separated CAR-T cells into three groups: resting, intermediate, and active (Fig. 5A). Both CD4^+^ and CD8^+^ CAR-T cells significantly increased the tumor response score with neuroblastoma stimulation (*P* < 2.2 × 10^−16^ by two-sided *t*-test) (Fig. 5B,C). Naive or resting T cell protein markers (e.g., CD62L, CD27, CD28, and CD127) were significantly enriched in the resting and intermediate groups (*P* < 1.27 × 10^−3^ by two-sided *t*-test), whereas T cell activation markers (e.g., CD137, CD25, and CD69) were significantly elevated in the active group (*P* < 5.58 × 10^−5^ by two-sided *t*-test) (Fig. 5D). Differential gene expression analysis identified 111, 54, and 337 highly expressed genes in the resting, intermediate, and active groups, respectively (Fig. 5E). In particular, differentially expressed genes in the intermediate group contained cell proliferation–related genes (e.g., *TOP2A*, *MKI67*, and *E2F2*). In contrast, the active group highly expressed ribosome binding genes (e.g., *EEF2* and *EIF6*) as well as genes encoding T cell activation cytokines (e.g., *IL2RA* and *CCL3*) (Trifilo et al. 2003; Liao et al. 2013). The active proliferation followed by ribosomal activation is consistent with the dynamic changes of CAR-T cells treated in patients (Li et al. 2021). PDCD1 (also known as PD-1 or CD279) is one of major inhibitory regulators of cytokine production and effector function and leads to T cell exhaustion (Gumber and Wang 2022). Because PD-1 expression was weakly elevated in the active group (*P* < 3.91 × 10^−6^ by two-sided *t*-test) (Fig. 5D), we asked whether the active group also displays molecular characteristics of T cell exhaustion. Interestingly, a cell cluster with the highest PD-1 expression displayed significant upregulation of tumor necrosis factor (TNF) production (e.g., *TNFAIP3* and *SELENOP3*) and endoplasmic reticulum (ER) stress (e.g., *DDIT3* and *HERPUD1*), which are known to contribute to the induction of T cell exhaustion (Fig. 5F; Beyer et al. 2016; Hurst et al. 2019). Furthermore, Gene Set Enrichment Analysis (GSEA) demonstrated that T cell exhaustion gene signatures were significantly elevated along the inferred tumor response score (Fig. 5G; Belk et al. 2022). Overall, these gene and surface protein expression patterns suggest that scIDST has the capacity to infer the extent of T cell activation and exhaustion.

Cell trajectory inference is a computational method to order cells based on transcriptional similarity and to characterize the molecular changes through pseudotemporal ordering (Cao et al. 2019). Next, we tested whether cell trajectory analysis also can infer T cell activation from the CITE-seq profiles using Monocle 3 (Cao et al. 2019). The Monocle 3–based pseudotime value showed different patterns from the scIDST-based score (Fig. 5C). However, several naive and resting markers (e.g., CD27, CD28, and CD127) were not differentially expressed through the pseudotime. In contrast, the pseudotime is significantly correlated with expression of cell proliferation–related genes (Pearson correlation coefficient = 0.545, *P* < 2.2 × 10^−16^) (Fig. 5C). The T cell exhaustion gene signatures were also not differentially expressed along the pseudotime (Fig. 5G). Furthermore, we also performed Monocle 3 in CD4^+^ and CD8^+^ CAR-T cells separately (Supplemental Fig. S4A,B). Similarly, naive and resting makers were not differentially expressed along the pseudotimes (Supplemental Fig. S4C). The pseudotimes were still ordered by the proliferation levels in both CD4^+^ and CD8^+^ CAR-T cells (Pearson correlation coefficient = 0.632 and 0.638 respectively, *P* < 2.2 × 10^−16^). In contrast, the antitumor response score by scIDST is more positively and negatively correlated with the expression of activated (e.g., CD137 and CD25) and naive T cell markers (e.g., CD62L and CD27), respectively. Taken together, our results indicate that scIDST shows superior performance in predicting antitumor response of CAR-T cells.

## Discussion

This study demonstrated that the weakly supervised deep learning can infer the varying pathological states (Figs. 1, 3) and the heterogeneous cellular responses to small molecules and pathogens (Figs. 4, 5) and is applicable to multiple independent scRNA-seq data sets (Fig. 2). The weak supervision is a new paradigm of machine learning that is optimal for large amounts of low-quality data labels, whereas other supervised learning methods (e.g., semisupervised learning) strongly rely on the accuracy of the labels in the training data sets. The weak supervision algorithm has been widely used for diagnosis in medical communities (Ratner et al. 2020) but requires heuristics (rules) that must be manually defined by users with their prior knowledge. However, it is challenging to determine optimal user-defined heuristics in the analysis of patient-derived single-cell sequencing data, owing to a limited knowledge about biological and pathological mechanisms. To solve this, we employed an automatic heuristic generator, Reef/Snuba (Varma and Ré 2018) and optimized this algorithm for the disease progression prediction.

Pseudotemporal ordering has been widely used to infer various dynamic biological processes (Trapnell et al. 2014; Adams et al. 2024). However, it heavily relies on an inferred cell trajectory dependent on a continuous set of patient-derived samples from latent to progressive stages in order to achieve sufficient accuracy (Tritschler et al. 2019). The weakly supervised deep learning can estimate disease progression levels without reconstruction of the cell trajectory and is capable of segregating disease-advanced cells from healthy and early-diseased stage of cells. In this study, the performance of scIDST was assessed in eight different scRNA-seq data sets of brain tissues and CAR-T cells but not in other tissues or atlas-scale data sets. However, the cross-data set applicability of scIDST gives rise to its potential for uncovering the shared mechanisms across different organs and multiple diseases. Taken together, the ability of scIDST to estimate disease status and drug response may assist in differential gene expression analysis of single-cell transcriptome data and may provide fascinating molecular insights of disease.

## Methods

### Supervised and weakly supervised deep learning

Our weakly supervised deep learning model is composed of three main steps: (1) autoencoder-mediated dimensional reduction, (2) generation of probabilistic labels, and (3) classification of diseased cells with a multilayered artificial neural network. The supervised deep learning model skips the probability labeling step and directly uses user-given binary labels for the classifier model. The Tensorflow Python library (v2.9.0) with Keras Tuner API (v1.1.2, https://github.com/keras-team/keras-tuner) was employed to implement deep learning (Abadi et al. 2016). In the following sections, technical descriptions of scIDST are provided.

#### Preparation of single-cell data and binary data labels

As input, scIDST requires (1) feature–barcode matrix and (2) binary data labels. Files of feature–barcode matrix (matrix.mtx.gz, barcodes.tsv.gz, and features.tsv.gz) can be generated by CellRanger aligner. scIDST package also provides an R script, *sgMatrix_table.R*, that can generate the feature–barcode matrix file set from Seurat object, which is a widely used R package for quality control, normalization, and data visualization (Hafemeister and Satija 2019). Binary data labels are manually created by users according to patient/donor information (e.g., 1 for PD patients, 0 for healthy donors) and saved in a comma-separated value (CSV) format.

#### Dimensional reduction by autoencoder: autoencoder.py

The algorithm consists initially of an autoencoder that is an artificial neural network to compress scRNA-seq data into a lower dimension (Hinton and Salakhutdinov 2006). The autoencoder is capable of capturing nonlinear relationships across data and is more appropriate for large complex data sets than other dimensionality reduction methods (e.g., PCA) (Hinton and Salakhutdinov 2006). Hyperparameters of the autoencoder are tunable by Keras tuner (https://github.com/keras-team/keras-tuner) to optimize the nonlinear dimensionality reduction of the input: the number of hidden layers (ranging from zero to 10 with increments of one, the number of nodes per hidden layer (ranging from zero to 1000 with increments of 200), and the number of nodes encompassing the latent space (ranging from 100 to 500 with increments of 100). These parameters are applied on the encoder as well as the decoder of the model. Additionally, a hyperbolic tangent activation function is employed on each layer of the autoencoder, except for the output layer of the decoder, which uses a sigmoid function. The model is trained on the normalized feature–barcode matrix with 10 epochs, using an optimizer function (e.g., Adam) to minimize the mean of the sum of the difference of the square between the predicted output and input (i.e., the mean squared error loss function). Once the optimized weights are calculated, the encoder of the model is employed to obtain a reduced representation of the input.

A Python script, *autoencoder.py*, takes the feature–barcode matrix as input and generates (1) the dimensionally reduced matrix file (CSV format) and (2) a directory storing the parameters of the autoencoder. The script also can perform the dimensionality reduction from a pretrained autoencoder.

#### Dimensional reduction by variational autoencoder: variational_autoencoder.py

As an alternative way for the dimensional reduction, we provide another Python script, *variational_autoencoder.py*. This script implements a variational autoencoder that employs probabilistic framework in latent space and less susceptible to overfitting than autoencoder (Kingma and Welling 2019). Similarly, this script takes the feature–barcode matrix as input and generates the dimensionally reduced matrix. Currently, scIDST does not support PCA as dimensional reduction methods, because principal components (PCs) separately generated from two different data sets cannot be directly compared; that is, PC1 on data set 1 and PC1 on data set 2 are generated from different variables.

#### Calculation of probabilistic labels: reef_analysis.py and convert_label.py

The conversion of the binary labels to probabilistic labels is implemented by Reef/Snuba algorithm (Varma and Ré 2018). Briefly, Reef/Snuba system first generates multiple “candidate” heuristics (decision trees) from a small portion of the dimensionally reduced single-cell data sets and the binary labels (Fig. 1B). The quality of each heuristic is then assessed by its performance: F1 score = [2 × (true positive)/(2 × true positive + false positive + false negative)] and diversity [1 − (Jaccard similarity between the assessed heuristic and the existing collection of best heuristics)]. The best heuristic is selected by the weighted average of these two criteria and added into the collection of heuristics. The Reef/Snuba performs these steps iteratively (about 50 times) and finally calculates the probabilistic labels by averaging outputs from the collection of best heuristics. Although the quality of each heuristic is assessed in Reef/Snuba, there is currently no method to evaluate the quality of the final collection of the heuristics.

In scIDST pipeline, 10% of the single-cell data set is randomly selected and used to develop the heuristics that thereafter assign the probabilistic labels to individual cells in the other 90% of the data set. This process is repeated at multiple times (e.g., 10 times), and the average of the probabilistic labels is used for training of subsequent classifier model. A Python script, *reef_analysis.py*, takes the dimensionally reduced data matrix (CSV format), the binary labels (CSV format), and a phenotype (e.g., disease and age), which users want to convert into probabilistic labels, as input. The output of this script is probabilistic labels of each repeat. Merging of the probabilistic labels in each phenotype and the calculation of average probabilistic labels are implemented by another script, *convert_label.py*, which finally generates a matrix of probabilistic labels in CSV format.

We note that the imbalanced data may affect the quality of the probabilistic labels. At least 20% of patient-derived cells is needed for proper estimation of the probabilistic labels. Thus, if the data set is skewed to specific class (e.g., healthy donor), we recommend under- and oversampling the skewed class before running *reef_analysis.py* (Supplemental Note).

#### Classification of diseased cells: classifier_analysis.py and tensor_analysis.py

The classifier model is a multilayered artificial neural network that can capture more complicated data patterns than single-layer network can (Abadi et al. 2016). The developed sequential model is constructed with seven tunable hyperparameters. It consists of an input layer and hidden layers, which, similar to the autoencoder model, varied in (1) number (two to 10 with increments of 1) and (2) nodes containing them (50 to 500 with increments of 50). Additionally, in order to avoid overfitting, a dropout layer, with (3) variable dropout rates of 0.1 from zero to 0.5, is introduced prior to the output layer. Although a softmax is used as activation function of the output layer, (4) multiple activation functions, such as the rectified linear unit, the sigmoid, and the hyperbolic tangent, are tested on the hidden layers. Finally, the model is trained with 20 epochs, using (5) various optimizers (e.g., Adam, SGD) and (6) learning rates (e.g., 1 × 10^−1^, 1 × 10^−2^, 1 × 10^−3^, 1 × 10^−4^, 1 × 10^−5^) to minimize (7) different loss functions (e.g., the mean squared error loss function, binary cross-entropy, categorial cross-entropy).

A Python script, *classifier_analysis.py*, is composed of two submodes: *train* and *predict*. The *train* mode performs the model training on the dimensionally reduced matrix and the probabilistic labels and gives (1) predicted scores of each phenotype (CSV format) and (2) a directory of the model parameters. If the “−l” option is selected, the script randomly selects a subset of data, which is used for the evaluation of the prediction performance but not used for the model training. The *predict* mode reads the dimensionally reduced matrix and performs the score calculation from a pretrained classifier model.

Another Python script, *tensor_analysis.py*, is an alternative command to construct the classification model, whose parameters (e.g., optimizers, loss function) can be determined by user. Similarly, the *train* mode requires the dimensionally reduced matrix and the probabilistic labels to output the predicted score and the model directory because the hyperparameter tuning is skipped. This is much faster than *classifier_analysis.py* and can be used to test the performance of weakly supervised learning.

Further questions and trouble shootings related to the weakly supervised deep learning models are shown in the Supplemental Notes.

### Preprocessing of single-cell RNA-seq

Single-cell transcriptome data of PD-derived brains and aged and healthy donors were preprocessed as described previously (obtained from the NCBI Gene Expression Omnibus [GEO; https://www.ncbi.nlm.nih.gov/geo/] under accession number GSE193688) (Adams et al. 2024). The count matrices of RNAs, antibody-derived tags (ADTs) and CAR binder library sequencing data were downloaded from GEO (GSE181437) (Tian et al. 2022). Single-cell RNA-seq data of idiopathic PD (SRP281977) (Smajić et al. 2022), putamen in PD (SRP291578) (Xu et al. 2023), epilepsy brain (SRP132816) (Velmeshev et al. 2019), brain tumor (SRP227039) (Bhaduri et al. 2020), AD (SRP291332) (Smith et al. 2022), and DHT-treated brain organoid (SRP344464) (Kelava et al. 2022) were downloaded from NCBI Short-Read Archive (SRA; https://www.ncbi.nlm.nih.gov/sra). The patient information was obtained from literature or SRA meta datasheet and used to make binary labels: namely, 0 (healthy) or 1 (disease), 0 (young) or 1 (aged), and 0 (female) or 1 (male). Raw sequence reads were aligned to human reference transcriptome (GRCh38, 2020-A) by CellRanger (v7.0.0) with default parameters. Quality control and cell clustering were then performed with Seurat (v4.0.0) in R (v4.0.5) (Hafemeister and Satija 2019; R Core Team 2023). First, we filtered out cells with (1) more than 7000 or less than 100 detected genes, (2) more than 20,000 or less than 500 total UMI count, and (3) >5% of mitochondria-derived reads from subsequent deep learning analysis. Then, the raw UMI count matrix was normalized to total UMI count and used to identify the top 2000 highly variable genes that were in turn used to detect cell pairs anchoring different scRNA-seq libraries. Using these anchoring cell pairs, all scRNA-seq libraries were merged and scaled. Dimensional reduction of the merged scRNA-seq libraries was performed by principal component analysis (PCA). All cells in the merged scRNA-seq libraries were further projected into two-dimensional UMAP space using one to 20 PCs. Graph-based clustering was then implemented with shared nearest neighbor method from first and 20th PCs and 0.8 resolution value (Supplemental Fig. S1B,C). We note that the inverse correlation between cluster size and the number of differentially expressed genes was detected regardless of the resolution value (Supplemental Fig. S1D). Cell types were determined per “island” by expression of cell type–specific markers as described previously (Tanaka et al. 2020; Adams et al. 2024). Differentially expressed genes were identified with more than 1.25-fold change and *P* < 0.05 by a two-sided unpaired *t*-test in each cell type or cluster. Significant GO terms in differentially expressed genes were identified by GOstats (v2.56.0) in R packages (Falcon and Gentleman 2007). The false-discovery rate (FDR) was calculated by adjusting the *P*-value with Benjamini–Hochberg correction using *p.adjust* function with method = “BH” option in R. Less than 0.05 FDR was defined as the statistical significance of the GO term. GSEA (v4.1.10) of was performed to genes sorted by Pearson correlation coefficients with the inferred disease progression score or pseudotime (Subramanian et al. 2005). Further details of each analysis were described in the Supplemental Methods.

### Software availability

The source code and Supplemental scripts for scIDST, written in Python or R, are available as Supplemental Code 1 and at GitHub (https://github.com/ytanaka-bio/scIDST). R scripts for the data preprocessing are also available as Supplemental Code 2 and at GitHub (https://github.com/ytanaka-bio/Wehbe_2024).

## Supplemental Material

Supplement 1

Supplement 2

Supplement 3

Supplement 4

Supplement 5
