# Supplementary material for: Inferring disease progression stages in single-cell transcriptomics using a weakly supervised deep learning approach
Source: Genome Res. 2025 Jan;35(1):135–46. doi: 10.1101/gr.278812.123 (PMC11789631; doi:10.1101/gr.278812.123)
Supplement: Supplement 1 [file Supplemental_Fig.pdf]

Supplemental Figures

Inferring disease progressive stages in single-cell transcriptomics using a weakly-supervised deep learning approach

Fabien Wehbe, Levi Adams, Jordan Babadoudou, Samantha Yuen, Yoon-Seong Kim, and Yoshiaki Tanaka

Table of Contents

Supplemental Figure 1. Conventional scRNA-seq analysis and application of supervised learning model to single-cell transcriptome profiles of Parkinson's disease patients

Supplemental Figure 2. Application of weakly-supervised deep learning model to Parkinson's disease patient-derived single-cell transcriptome profiles

Supplemental Figure 3. Application of pre-trained weakly-supervised models to other independent single-cell datasets

Supplemental Figure 4. Inference of $CD4^+$ and $CD8^+$ CAR-T cell activation dynamics to neuroblastoma

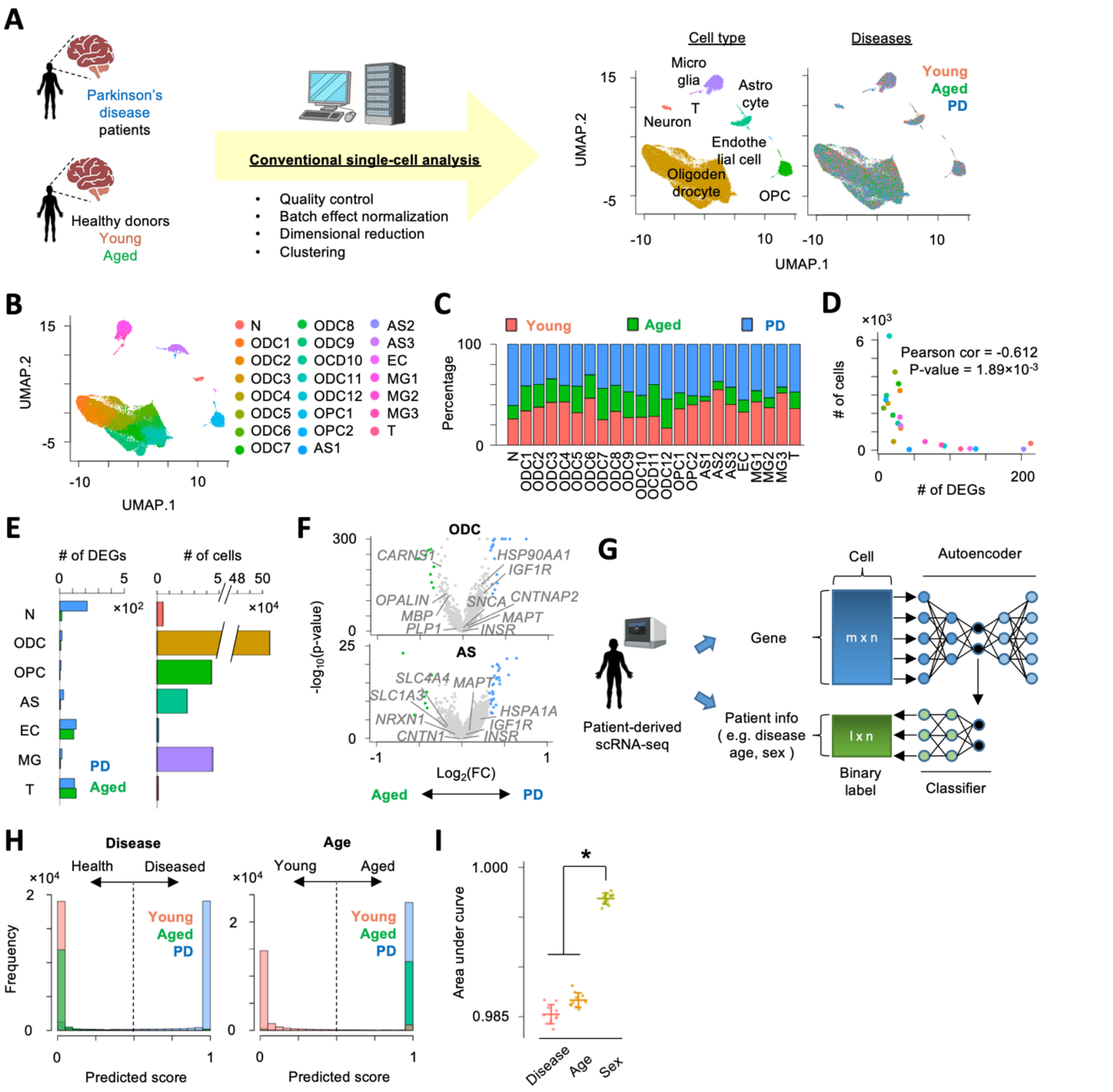

Supplemental Figure 1. Conventional scRNA-seq analysis and application of supervised learning model to single-cell transcriptome profiles of Parkinson's disease patients

A. Conventional single-cell analysis clusters cells into cell types, but not into disease**. B.** UMAP plots of single cells colored by clusters. **C.** Composition of origins of cells in each cluster. **D.** Inverse correlation between the number of differentially-expressed genes and the number of cells in each cluster. **E.** Comparison between (left) the number of differentially-expressed genes (DEGs) between Parkinson's disease patient and age-matched healthy donor and (right) the number of cells in each cell type. **F.** Volcano plots showing differential expression between PD patients and age-matched donors **G.** Supervised deep learning model to discriminate diseased and healthy cells. **H.** Histogram of prediction score (output of deep learning model) of disease and age. **I.** Comparison of area under curve in the prediction of disease, age, and sex. Error bar represents standard deviation (n=10). * $p<0.05$ by two-sided *t*-test.

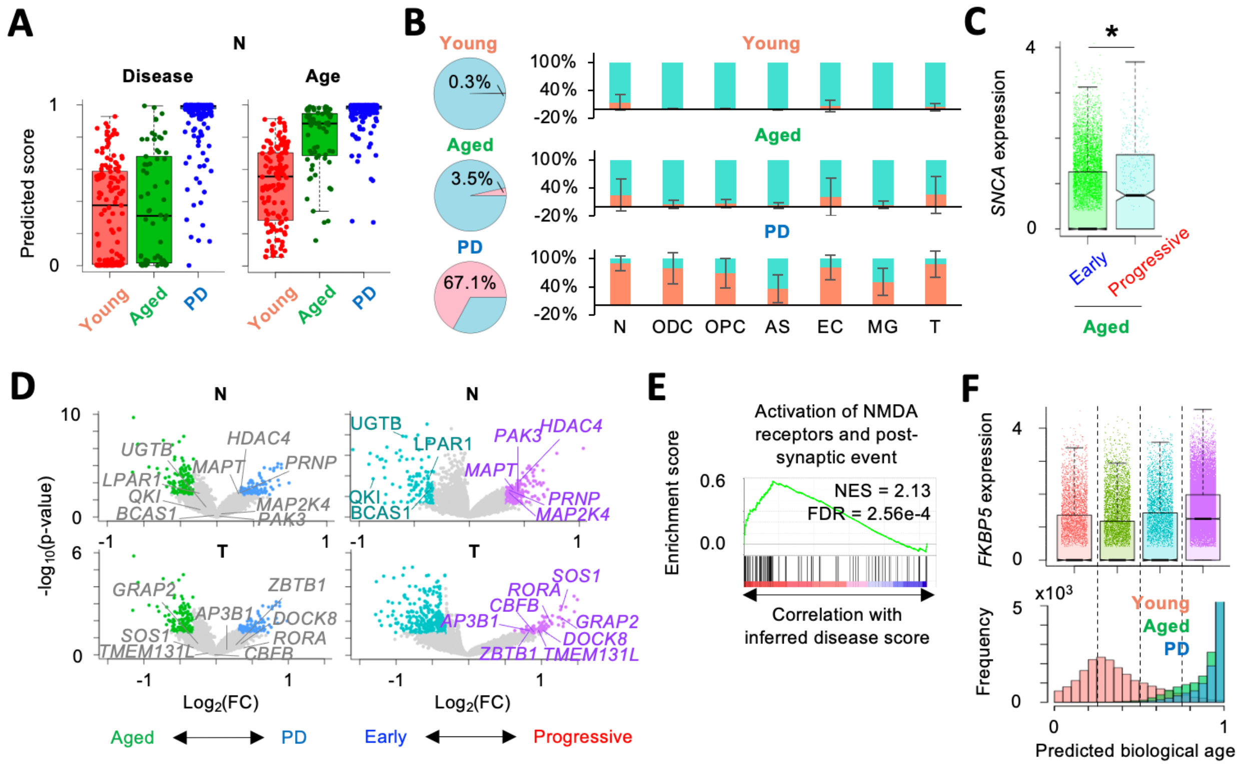

Supplemental Figure 2. Application of weakly-supervised deep learning model to Parkinson's disease patient-derived single-cell transcriptome profiles

A. Comparison of the inferred disease progressive levels and biological ages in neurons across healthy young, aged donors, and Parkinson's disease patients. **B.** Pie charts showing frequency of disease progressive cells. The frequency in each cell type is shown in right panel. Error bars represent standard deviation. **C.** Comparison of *SNCA* expression between early/healthy and disease progressive cells in aged brain. **D.** Volcano plot showing differential expression in neurons and T cells (left) between PD patients and healthy aged donors and (right) between disease progressive stage and early/heathy stage. **E.** Activation of NMDA receptor pathway over the inferred disease process. **F.** Correlation between predicted biological age and *FKBP5* expression. Cells are separated into four groups by predicted biological age. Comparison of *FKBP5* expression across four groups and Histogram of predicted biological age are shown in top and bottom, respectively.

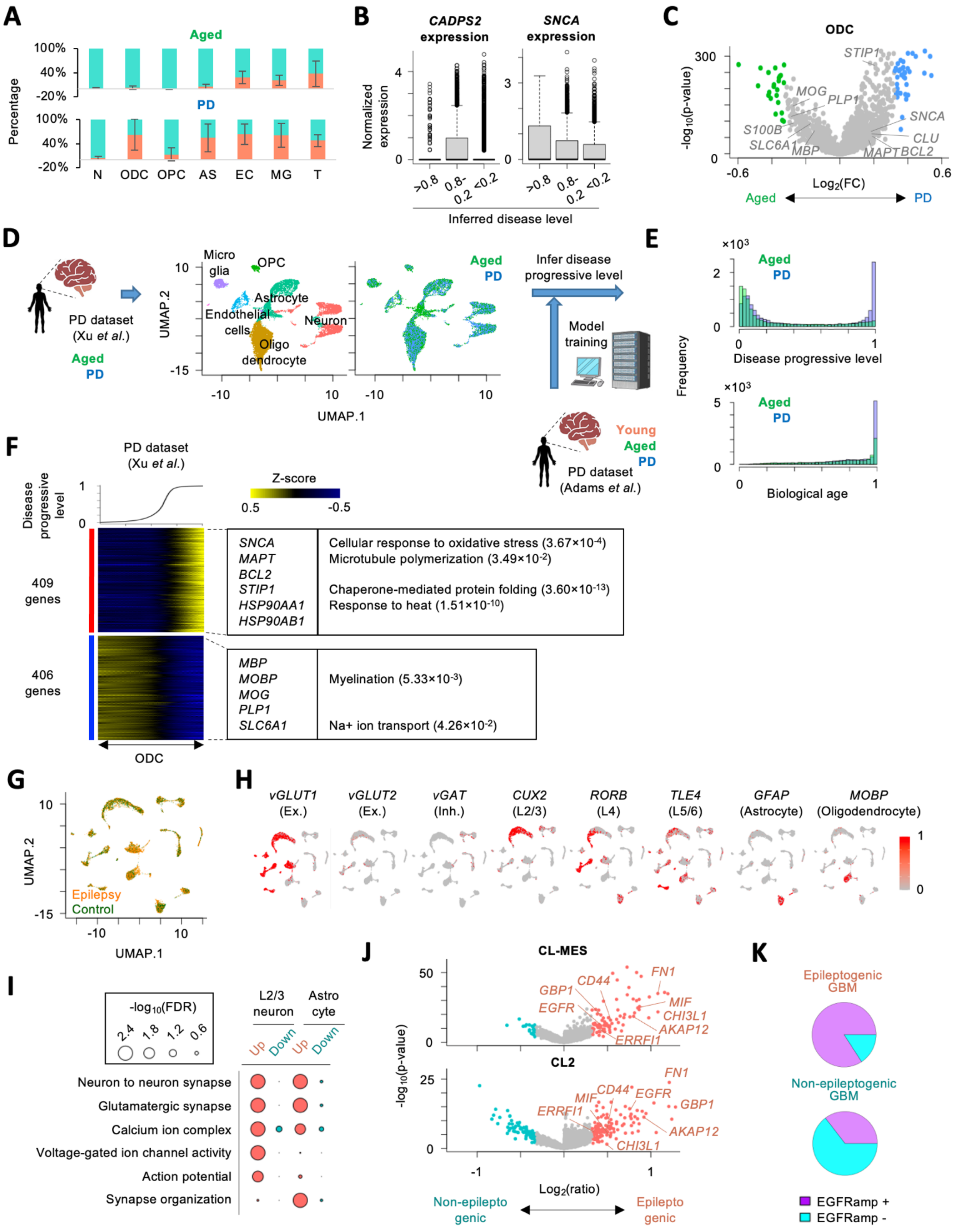
A
Aged
PD
Percentage
100%
40%
-20%
N ODC OPC AS EC MG T
B
CADPS2 expression
SNCA expression
Normalized expression
>0.8 0.8-0.2 <0.2
Inferred disease level
C
ODC
-log10(p-value)
STIP1
MOG
PLP1
S100B
SLC6A1
MBP
SNCA
CLU
MAPT
BCL2
Log2(FC)
Aged
PD
D
PD dataset (Xu et al.)
Aged
PD
Micro glia
OPC
Astrocyte
Endothelial cells
Neuron
Oligo dendrocyte
UMAP.2
UMAP.1
Infer disease progressive level
Model training
Young
Aged
PD
PD dataset (Adams et al.)
E
Frequency
Disease progressive level
Biological age
F
PD dataset (Xu et al.)
Disease progressive level
Z-score
409 genes
406 genes
SNCA
MAPT
BCL2
STIP1
HSP90AA1
HSP90AB1
Cellular response to oxidative stress (3.67×10-4)
Microtubule polymerization (3.49×10-2)
Chaperone-mediated protein folding (3.60×10-13)
Response to heat (1.51×10-10)
MBP
MOBP
MOG
PLP1
SLC6A1
Myelination (5.33×10-3)
Na+ ion transport (4.26×10-2)
ODC
G
UMAP.2
UMAP.1
Epilepsy
Control
H
vGLUT1 (Ex.)
vGLUT2 (Ex.)
vGAT (Inh.)
CUX2 (L2/3)
RORB (L4)
TLE4 (L5/6)
GFAP (Astrocyte)
MOBP (Oligodendrocyte)
I
-log10(FDR)
2.4 1.8 1.2 0.6
L2/3 neuron
Astro cyte
Up Down Up Down
Neuron to neuron synapse
Glutamatergic synapse
Calcium ion complex
Voltage-gated ion channel activity
Action potential
Synapse organization
J
CL-MES
CL2
FN1
CD44
GBP1
EGFR
MIF
CHI3L1
AKAP12
ERRFI1
-log10(p-value)
Log2(ratio)
Non-epilepto genic
Epilepto genic
K
Epileptogenic GBM
Non-epileptogenic GBM
EGFRamp +
EGFRamp -

Supplemental Figure 3. Application of pre-trained weakly-supervised models to other independent single-cell datasets

A. Frequency of the inferred disease progressive cells in each cell type of Smajic *et al.* dataset. Error bars represent standard deviation. **B.** Comparison of (L) *CADPS2* and (R) *SNCA* expression across three groups that were classified by the inferred disease progressive level. **C.** Volcano plot showing differential expression in oligodendrocyte between PD patients and healthy aged donors in Smajic *et al.* dataset. **D.** Strategy to estimate Parkinson's disease progressive level by the pre-trained model in another scRNA-seq dataset from different brain area. **E.** Histogram of predicted score of disease progressive level and biological age. **F.** Heatmap showing genes, whose expression is highly correlated with inferred disease progressive score (Genes with Pearson correlation > 0.1). Oligodendrocyte cells are sorted by the disease progressive score. Heatmap color represents z-score-normalized gene expression. Representative genes and GO terms are shown in right panel. Statistical significance of GO terms is shown by false discovery rate. **G.** UMAP plot of single cells colored by epilepsy patients (orange) and healthy control donors (darkgreen). **H.** UMAP plot showing representative cell type-specific gene expression in epilepsy patients and healthy donors-derived scRNA-seq dataset. **I.** Representative GO terms overrepresented in differentially-expressed genes between epileptic and non-epileptic cells. Circle size represents $-\log_{10}$(FDR). **J.** Volcano plots showing differential expression between epileptogenic and non-epileptogenic GBM cells in CL-MES and CL2 cluster. Significantly-upregulated ERK1/2 cascade-related genes are also shown. **K.** Pie chart showing ratio of cells with EGFR amplification in the inferred epileptogenic and non-epileptogenic GBM cells.

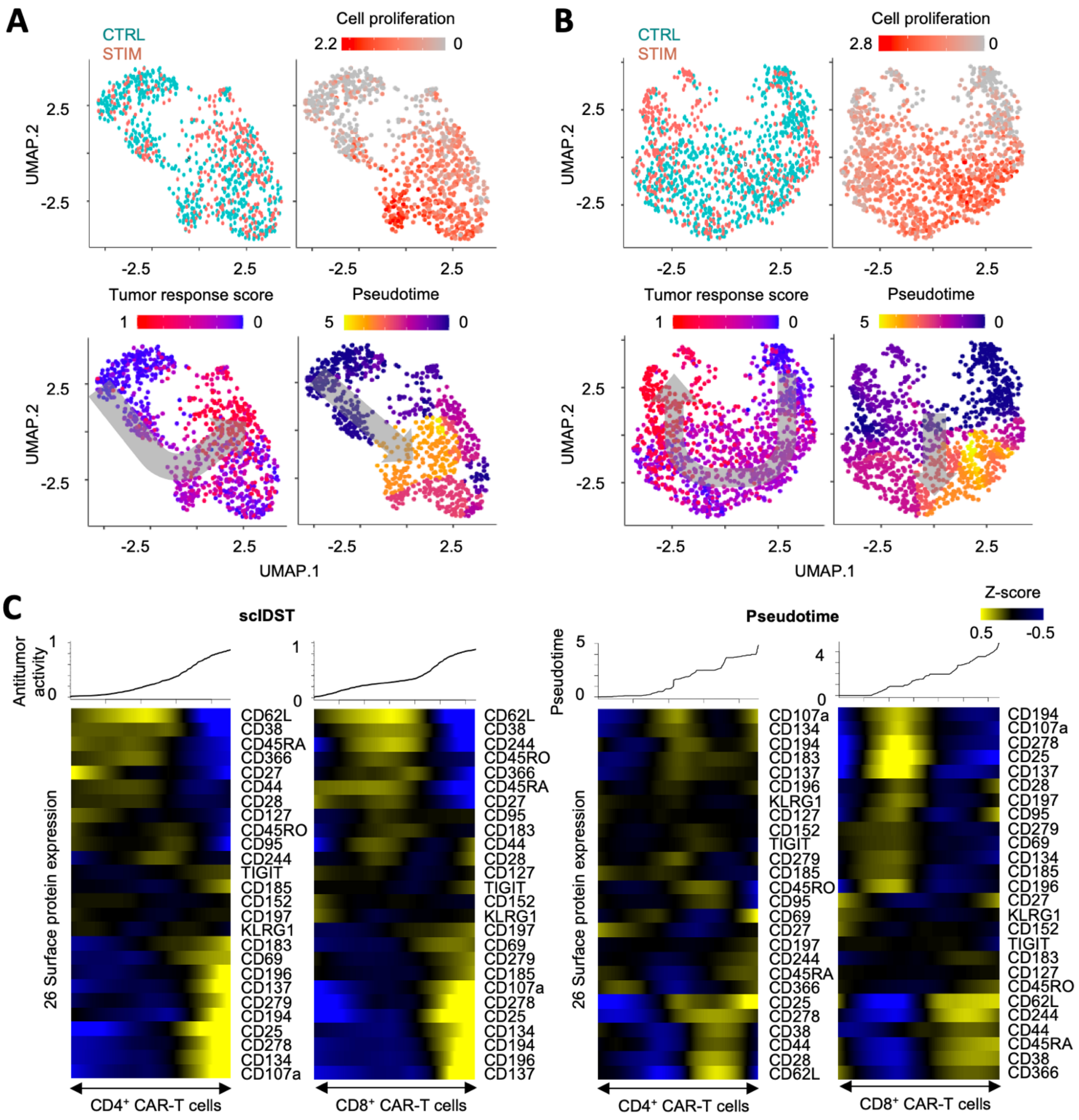

Supplemental Figure 4. Inference of CD4+ and CD8+ CAR-T cell activation dynamics to neuroblastoma

A-B. UMAP plots of **(A)** CD4+ and **(B)** CD8+ individual CAR-T cells colored by the stimulation conditions (top left), cell proliferation-related gene expression (top right), the inferred antitumor activity (bottom left), and pseudotime (bottom right). **C.** Heatmap showing 26 surface protein expression dynamics in CD4+ and CD8+ CAR-T cells. CAR-T cells were sorted by (left) scIDST or (right) pseudotime. Heatmap color represents z-score-normalized protein expression.
