# Supplementary material for: Inferring disease progression stages in single-cell transcriptomics using a weakly supervised deep learning approach
Source: Genome Res. 2025 Jan;35(1):135–46. doi: 10.1101/gr.278812.123 (PMC11789631; doi:10.1101/gr.278812.123)
Supplement: Supplement 2 [file Supplemental_Note.pdf]

Supplemental Note

Inferring disease progressive stages in single-cell transcriptomics using a weakly-supervised deep learning approach

Fabien Wehbe, Levi Adams, Jordan Babadoudou, Samantha Yuen, Yoon-Seong Kim, and Yoshiaki Tanaka

See also our Wiki at GitHub repository (https://github.com/ytanaka-bio/scIDST/wiki)

1. Does imbalanced data affect output?

The dataset (GEO: GSE193688) (Adams et al. 2024) contain 14 PD patients and 9 young and 8 aged healthy donors-derived scRNA-seq libraries. To test the effects of imbalanced data, we gradually remove PD samples (from 14 samples to 1 sample), and performed Reef/Snuba. We found that the probabilistic labels were dramatically decreased, when the number of patient-derived cells was less than 20% (removing more than half of PD samples). If the number of patient-derived cells is more than 20%, the probabilistic labels were very similar.

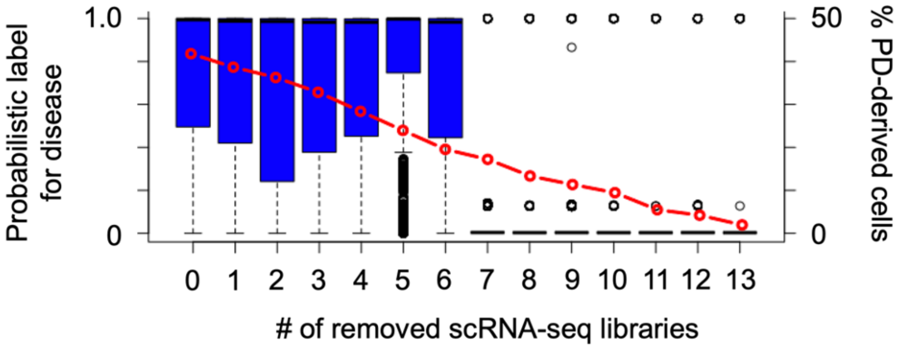

Appendix 1. Distribution of the probabilistic labels in PD patient-derived cells. The calculation of the probabilistic labels was performed by gradually removing PD samples in Adams et al. 2024 dataset. The percentage of PD patient-derived cells is shown by red line.

The dataset imbalance is one of the issues in the integrative analysis of single-cell data (Maan et al. 2024). If the dataset is skewed to specific class, we recommend under or oversampling the skewed class before running Reef/Snuba. For example, if patient-derived cells are less than 20%, please under or oversample cells from healthy donors or patients respectively, and increase the ratio of the minority groups. This under and oversampling improves the performance of Reef/Snuba probabilistic label calculation.

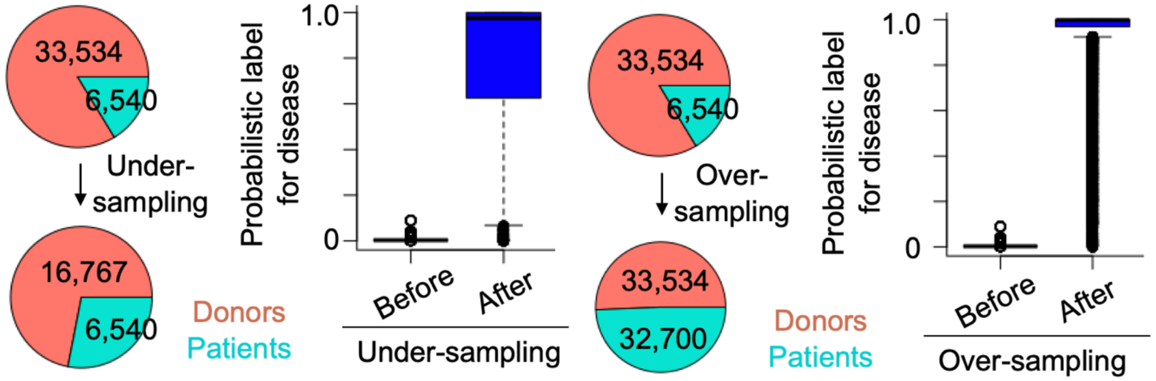

Appendix 2. Improvement of the probabilistic label calculation by under and oversampling. Given dataset with less than 20% of patient-derived cells, the under or oversampling of cells from healthy donors or patients respectively improved the performance of probabilistic label calculation.

2. What is a difference between the classifier's output and the probability labels?

Reef/Snuba algorithm calculates the probabilistic labels by agreement and disagreement of decision trees that are iteratively generated and pruned to fit a small portion of datasets. Therefore, the output from Reef/Snuba is relatively rough to that from subsequent ANN classifier. In addition, although the decision tree is faster and applicable for various types of datasets, ANN is more suitable to model complex relationships. Therefore, we recommend using the output of ANN classifier rather than the probabilistic labels from Reef/Snuba for subsequent analysis.

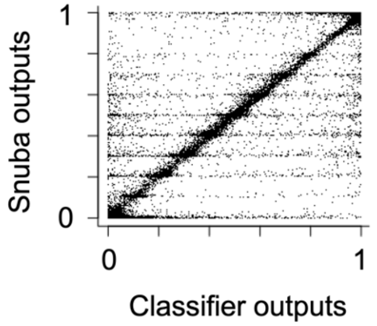

Appendix 3. Comparison of classifiers output and Reef/Snuba output.

3. How is batch effect correction?

By comparing the distribution of individual cells across different patient/donor samples, we demonstrated that the removal of the batch effect was comparable with that in Seurat dimension-reduced data. UMAP plots represent similar distribution of individual cells across patient/donor samples. These results indicates that the batch effect is trivial level in our autoencoder-based dimension-reduced data. Variational autoencoder (VAE) is an alternative dimensionality reduction method that is probabilistic mapping and less susceptible to

overfitting than autoencoder. Like Seurat and autoencoder, the VAE-based dimension-reduced data displayed limited batch effects.

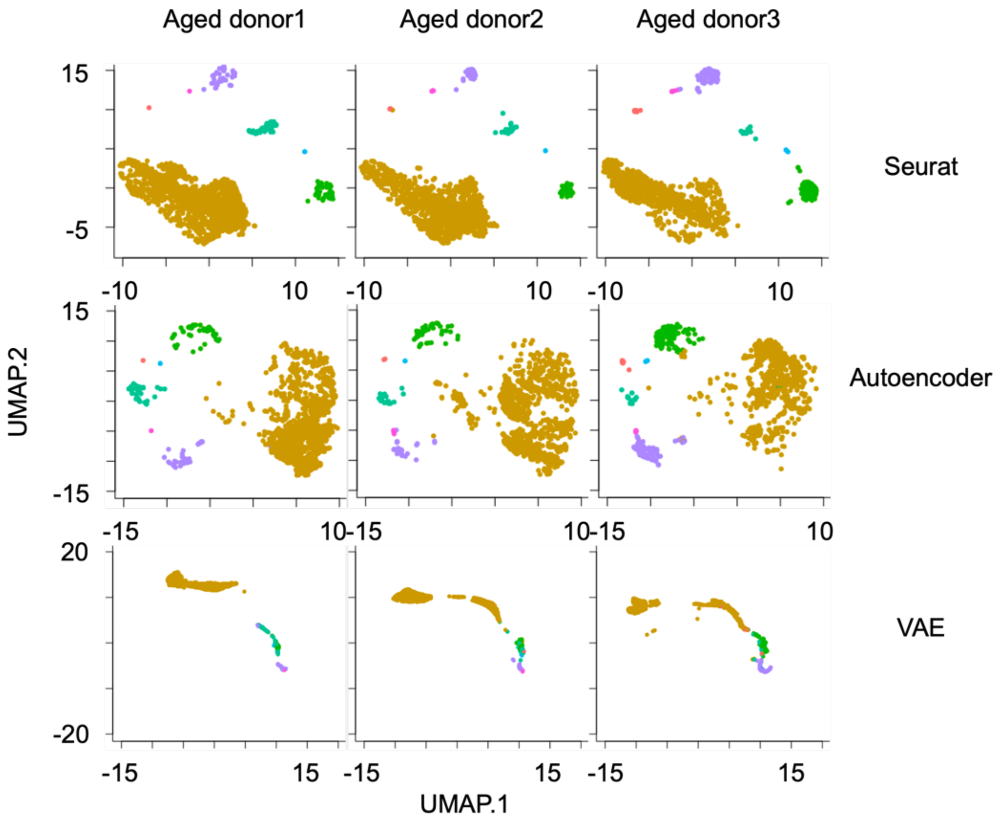

Appendix 4. Comparison of the batch effects by three dimension-reduced data: Seurat, autoencoder, and variational autoencoder (VAE). UMAP plots of individual cells from distinct patient/donor are shown.

4. <u>Does ratio of an initial dataset for Reef/Snuba algorithm affect the probabilistic labels?</u>

The probabilistic labels are calculated from a small portion (10% default, can be adjusted by -v option) of the datasets. We expect the bimodal distribution of disease level (healthy/early vs progressive), and test how this bimodality is affected by the ratio of the initial dataset. The bimodality was with low percentage of the initial datasets (10 ~ 50%), but disappeared when the ratio of the initial datasets were increased (70% ~). We noticed that the most of the probabilistic labels become 1 or 0 (similar with supervised learning), if we used high portion of the initial datasets. Therefore, we recommend that the ratio of the initial datasets should be set as small percentage (10~50%) for the weak supervision.

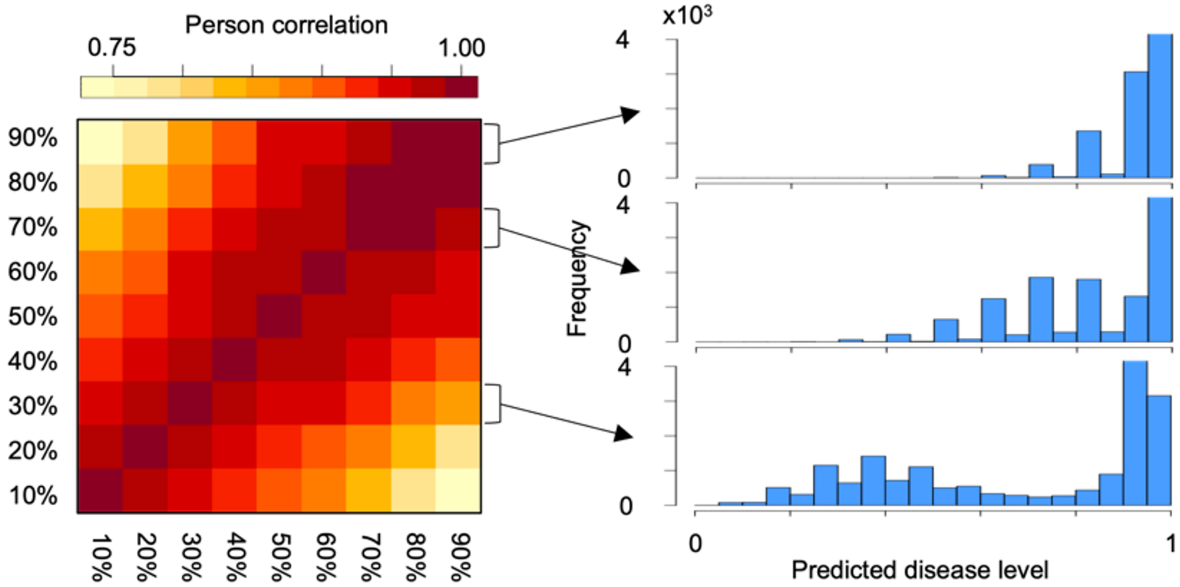

Appendix 5. Comparison of Reef/Snuba outputs with different ratio of initial datasets. The ratio was tested from 10% and 90%. (L) Heatmap represents Person correlation of Reef/Snuba outputs. (R) Histograms represents 3 representative Reef/Snuba outputs.

5. Does scIDST show better performance than other cell scoring tools?

We first tested scPred that calculates conditional class probabilities of belonging to a given cell states/subtypes by supervised support vector machine (Alquicira-Hernandez et al. 2019). Although the predicted disease levels were significantly higher in PD patient-derived cells than those from healthy donors ($p<2.2\times10^{-16}$ by two-sided *t*-test), significant difference of *SNCA* expression was not identified in the inferred disease progressive cells (Appendix 6A). In contrast, *FKBP5* gene expression was clearly elevated along the predicted biological age (Appendix 6B). Comparative analysis with scIDST revealed that the predicted biological ages were similar between scPred and scIDST, whereas the correlation of disease levels are very weak between two methods (Appendix 6C). Nearest neighbor graph is an alternative approach to predict cell-to-cell relationships (Baran et al. 2019), and continuous cell state transition (Dann et al. 2022). To assess its performance, we also tested Milo algorithm that implemented differential abundance testing by assigning individual cells into nodes on a *k*-nearest neighbor graph (Dann et al. 2022). Milo identified the nodes with differential abundance of PD patient/healthy donor-derived cells or aged/young donor-derived cells in neurons, oligodendrocytes, astrocytes, and microglia (Appendix 6D). However, a substantial number of differentially-expressed genes in these nodes were identified only in neurons or oligodendrocytes (Appendix 6E). Although the decline of myelin is one of pathological features of PD (Dean et al. 2016), there was no significant difference in the myelination-associated gene expression (e.g. *MBP*, *MOBP*) (Appendix 6F). In contrast, similarly with scIDST and scPred, significant elevation of *FKBP5* expression with aging was successfully detected. Taken together, these results indicated that our weakly-supervised deep learning displayed superior or comparable performance to the existing tools in the inference of disease progression and biological aging.

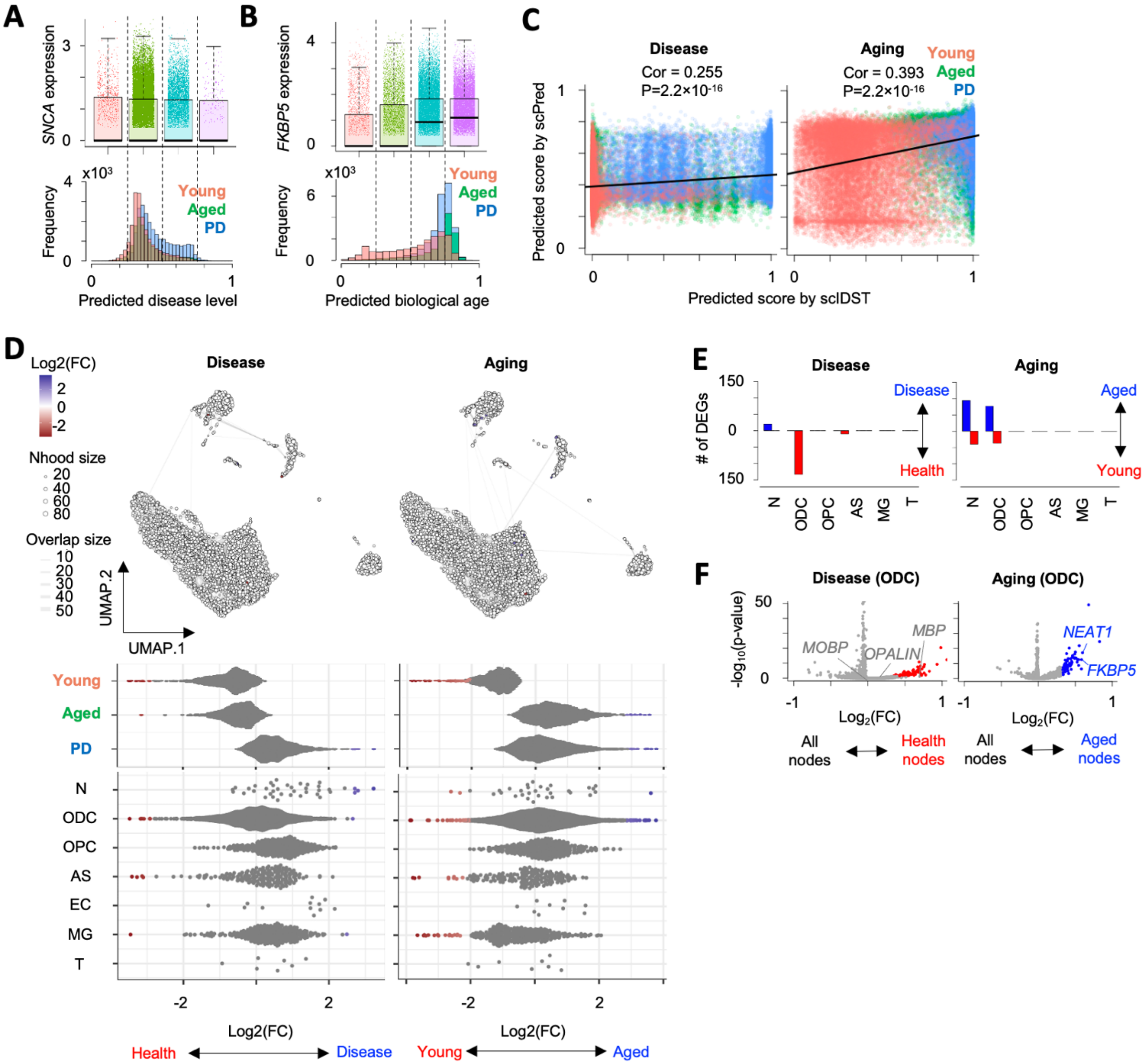

Appendix 6. Application of other cell scoring tools to Parkinson's disease patient-derived single-cell transcriptome profiles. A-B. Correlation **(A)** between scPred-predicted disease progressive level and *SNCA* expression and (**B)** between scPred-predicted biological age and *FKBP5* expression across four groups. **C.** Comparison of predicted disease progressive level and biological age between scIDST and scPred. Person correlation coefficient and p-value are shown. **D.** Neighborhood graphs by Milo differential abundance testing. Beeswarm plot of $\log_2$(fold change) by (L) disease and (R) aging in each neiborhood node is also shown. Nodes with significant differential abundance (FDR < 0.1) are shown by blue or red colors. **E.** The number of differentially-expressed genes in nodes with differential abundance of (L) PD patient-derived cells and (R) aged donor-derived cells. **F.** Volcano plots showing differential gene expression in nodes in with enrichment of (L) healthy donor-derived ODCs and (R) aged donor-derived ODCs.

Reference

Adams L, Song MK, Yuen S, Tanaka Y, Kim YS. 2024. A single-nuclei paired multiomic analysis of the human midbrain reveals age- and Parkinson's disease-associated glial changes. *Nat Aging* **4**: 364-378.

Alquicira-Hernandez J, Sathe A, Ji HP, Nguyen Q, Powell JE. 2019. scPred: accurate supervised method for cell-type classification from single-cell RNA-seq data. *Genome Biol* **20**: 264.

Baran Y, Bercovich A, Sebe-Pedros A, Lubling Y, Giladi A, Chomsky E, Meir Z, Hoichman M, Lifshitz A, Tanay A. 2019. MetaCell: analysis of single-cell RNA-seq data using K-nn graph partitions. *Genome Biol* **20**: 206.

Dann E, Henderson NC, Teichmann SA, Morgan MD, Marioni JC. 2022. Differential abundance testing on single-cell data using k-nearest neighbor graphs. *Nat Biotechnol* **40**: 245-253.

Dean DC, 3rd, Sojkova J, Hurley S, Kecskemeti S, Okonkwo O, Bendlin BB, Theisen F, Johnson SC, Alexander AL, Gallagher CL. 2016. Alterations of Myelin Content in Parkinson's Disease: A Cross-Sectional Neuroimaging Study. *PLoS One* **11**: e0163774.

Maan H, Zhang L, Yu C, Geuenich MJ, Campbell KR, Wang B. 2024. Characterizing the impacts of dataset imbalance on single-cell data integration. *Nat Biotechnol* doi:10.1038/s41587-023-02097-9.
