# Supplementary material for: Inferring disease progression stages in single-cell transcriptomics using a weakly supervised deep learning approach
Source: Genome Res. 2025 Jan;35(1):135–46. doi: 10.1101/gr.278812.123 (PMC11789631; doi:10.1101/gr.278812.123)
Supplement: Supplement 3 [file Supplemental_Methods.pdf]

Supplemental Note

Inferring disease progressive stages in single-cell transcriptomics using a weakly-supervised deep learning approach

Fabien Wehbe, Levi Adams, Jordan Babadoudou, Samantha Yuen, Yoon-Seong Kim, and Yoshiaki Tanaka

Table of Contents

1. Assessment of binary classification models by supervised deep learning
2. Application of weakly-supervised deep learning models to single-cell transcriptome profiles of Parkinson's disease
3. Application of existing cell scoring tools to predict disease progression and biological aging
4. Application of weakly-supervised deep learning models to single-cell transcriptome profiles of epilepsy and GBM patients
5. Application of weakly-supervised deep learning models to single-cell transcriptome profiles of Alzheimer's disease
6. Application of weakly-supervised deep learning models to single-cell transcriptome profiles of DHT-treated brain organoids
7. Application of weakly-supervised deep learning models to CITE-seq of CAR-T cells

1. Assessment of binary classification models by supervised deep learning

The feature-barcode matrix of PD patients and healthy young and aged donors (GSE193688) (Adams et al. 2024) was first dimensionally reduced by *autoencoder.py.* The classifier model was then trained and assessed by the output of the autoencoder and the binary labels *classifier_analysis.py* with "-l" option. Briefly, 20% of the dataset was randomly partitioned as test dataset. The remaining dataset was further randomly divided into training and validation dataset (80% and 20%, respectively). During the training step, the model parameters were initially fit on the training dataset, and turned by the validation dataset. True positive rates and false positive rates were calculated in each category (disease, age, and sex) with test dataset at various threshold setting. Area under the curve of receiver operating characteristic curve was finally used to assess the performance of the binary classification. We repeated the above steps at 10 times and statistically compare the prediction performance across disease, age, and sex (Figure S1I).

2. Application of weakly-supervised deep learning models to single-cell transcriptome profiles of Parkinson's disease

We trained the weakly-supervised deep learning model from a single-cell gene expression of PD patients and healthy young and aged donors (GSE193688) (Adams et al. 2024). Briefly, probabilistic labels were calculated in disease and age using the dimensionally-reduced matrix (the output of *autoencoder.py*) and the binary labels. Then, the classifier model was trained by the dimensionally-reduced matrix and the probabilistic labels. Using the trained model, we calculated scores of "disease progression" and "biological age" in three PD single-cell transcriptomic datasets (GSE193688) (Adams et al. 2024), SRP281977 (Smajic et al. 2022) and SRP291578 (Xu et al. 2023)). Individual cells were then separated into disease progressive and early-staged/healthy cells by 0.8 cutoff of the inferred disease progressive scores (Figure 1D). Differentially-expressed genes were identified by comparing gene expression profiles between disease progressive and early-staged/healthy cells in PD patients (Figure 1F) or age-matched donors (Figure S2C). Cells were also separated by the inferred biological ages into four groups with 0.25, 0.5, and 0.75 thresholds. Expression of a relevant age-dependent gene, *FKBP5*, was compared across these groups (Figure S2D). The correlations between the inferred disease progressive levels and gene expression were calculated by *cor* function with method="spearman" option in R. Significant correlation was defined as more than 0.1 or less than -0.1 with $p<2.2\times10^{-16}$ (Figure 2C). GO analysis was performed in the differentially-expressed genes or the correlated genes using GOstats R package (Falcon and Gentleman 2007).

3. Application of existing cell scoring tools to predict disease progression and biological aging

scPred (v1.9.2) and Milo (v1.10.0) were performed according to their instructions (Alquicira-Hernandez et al. 2019; Dann et al. 2022). Briefly, in scPred, the model training was first implemented with *trainModel* by selecting disease state (disease or healthy) or aging (aged or young). Subsequently, the probabilities to disease and aging were calculated by *scPredict* function with default parameters. In Milo, we first converted the Seurat object into SingleCellExperiment object, and then changed it to Milo object. *K*-nearest neighbor graph was constructed by *buildGraph* function with "k=10, d=20" options. Representative cells in each node was defined by *makeNhoods* function with "prop = 0.1, k = 10, d=20, refined = TRUE" options. After counting cells in each neighborhood across samples by *countCells* function, the neighborhood connectivity was computed by *calcNhoodDistance* function with

"d=20" option. Finally, the differential abundance of disease state (disease or healthy) or aging (aged or young) was estimated by *testNhoods* function.

4. Application of weakly-supervised deep learning models to single-cell transcriptome profiles of epilepsy and GBM patients

The weakly-supervised deep learning model was trained from a single-cell dataset of epilepsy patients and healthy donors (SRP132816) (Velmeshev et al. 2019). We set threshold of the inferred epileptogenic scores as 0.5, since almost all of cells from healthy donors were less than this cutoff (Figure 2E). The single-cell data of epilepsy patients and healthy donors was projected into UMAP dimension as described above (Figure 2F). Cell types were determined by cell-type specific markers. For example, L2/3 neurons were determined by co-expression of *vGLUT1* (*SLC17A7*) and *CUX2*, whereas astrocytes are by *GFAP*. Differentially expression analysis and GO analysis between epileptogenic and non-epileptogenic cells was performed in L2/3 neuron and astrocyte cluster, in which vast majority epileptogenic cells were observed (Figure S3I).

Using the trained model, we inferred epileptogenic levels in a single-cell dataset of GBM patients (SRP227039) (Bhaduri et al. 2020). The clusters were labeled by enrichment of markers for three GBM molecular subtypes: PN: proneural, CL: classical, and MES: mesenchymal (Verhaak et al. 2010; Wang et al. 2017). using GSEA software (v4.1.10) (Subramanian et al. 2005). A gene set is considered enriched for a certain cluster if its resulting false discovery rate (FDR) adjusted p-value was equal to or less than 0.05. Then, epileptogenic GBM cells were defined as more than 0.5 scores (Figure 2G). Enrichment of epileptogenic GBM cells were analyzed in each Seurat-based cluster. Differentially expression analysis and GO analysis between epileptogenic and non-epileptogenic GBM cells were performed in CL-MES and CL3 clusters, in which epileptogenic GBM cells were enriched (Figure 2H and S3J). EGFR amplification status in each patient was obtained from the literature (Figure S3K) (Bhaduri et al. 2020).

5. Application of weakly-supervised deep learning models to single-cell transcriptome profiles of Alzheimer's disease

The weakly-supervised deep learning model was trained by single-cell transcriptome profiles of Braak Stage VI (SRX9446250 and SRX9446251) and non-AD controls (SRX9446233, SRX9446234, SRX9446236, and SRX9446244) (Smith et al. 2022). The trained model was then used to infer disease progression levels of individual cells in AD patients with Braak Stage I (SRX9446247, SRX9446248, SRX9446249, SRX9446252, SRX9446253, SRX9446254, SRX9446255, and SRX9446256), III/IV (SRX9446237, SRX9446238, SRX9446240, and SRX9446245), and V (SRX9446239, SRX9446241, SRX9446243, and SRX9446246). The percentages of pTau and Aβ-positive cells/area in each patient were obtained from the literature (Smith et al. 2022) and compared with average of the inferred disease progression scores (Figure 3D). The correlations between the inferred disease progressive levels and gene expression were calculated by *cor* function with method="spearman" option in each cell type. Significant correlation was defined as more than 0.1 or less than -0.1 with $p<2.2\times10^{-16}$ (Figure 3E and 3F).

6. Application of weakly-supervised deep learning models to single-cell transcriptome profiles of DHT-treated brain organoids

The weakly-supervised deep learning model was trained by a single-cell data of DHT- and mock-treated brain organoids (SRP344464) (Kelava et al. 2022). The threshold of cellular

response to DHT was set as 0.55 (Figure 4A). After UMAP projection by Seurat, neuron (N1-5) and glia clusters (G1-11) were defined by expression of *STMN2* and *SOX2*, respectively. Neurons were further separated into excitatory (*vGLUT1* (*SLC17A7*)$^+$ or *vGLUT2* (*SLC17A6*)$^+$), inhibitory (*vGAT* (*SLC32A1*)$^+$) and non-committed neurons (*vGLUT1*$^-$, *vGLUT2*$^-$, and *vGAT*$^-$). Glia cells were separated into radial glia (*COL4A5*$^+$), dividing radial glia (*TOP2A*$^+$), and basal radial glia (*PTN*$^+$) (Figure 4C). The ratio of DHT-responded cells was compared across these subtypes (Figure 4D). Differentially-expressed genes (fold change > 1.25 and p<0.05 with two-sided *t*-test) between DHT- and non-responded cells and between DHT- and mock-treated cells (global comparison between DHT- and mock-treated organoids) was identified in N3 and G5 cluster (Figure 4E).

7. Application of weakly-supervised deep learning models to CITE-seq of CAR-T cells

Gene and protein expression matrices were obtained from NCBI GEO database (GSE181437) (Tian et al. 2022). First, singlet, doublet, and negative cells were identified from ADT matrix with *HTODemux* function in Seurat (Doublet cells were removed from the subsequent analyses) (Hafemeister and Satija 2019). The ADT matrix was then normalized and denoised by dsb R library (v1.0.2) (Mule et al. 2022). Briefly, the ADT count matrix of singlet and negative cells were inputted in *DSBNormalizeProtein* function as *cell_protein_matrix* and *empty_drop_matrix* parameter, respectively. Mouse IgG antibodies were used as isotype controls. Finally, the normalized ADT matrix was combined with RNA count matrix and used for the training of the weakly-supervised deep learning models. CAR-T cells were identified by the presence of CAR binder library sequences.

The normalized RNA and ADT matrices were also used for cell trajectory analysis. Briefly, the normalized RNA data slot in Seurat object was combined with the normalized ADT matrices with *rbind* function. Subsequently, the Seurat object was converted into Monocle 3 object using *as.cell_data_set* function in SeuratWrappers R package (v0.3.0). Cell trajectory graph was then constructed by *learn_graph* function in Monocle 3 R package (v0.2.3.0) (Cao et al. 2019). Finally, pseudotime was calculated by *order_cells* function by choosing one cluster, where the percentage of non-stimulated CAR-T cells is the highest, as root cells. The association of pseudotime with cell proliferation was analyzed by Pearson correlation between pseudotime and average expression of three major cell proliferation signatures: *TOP2A*, *MKI67*, and *E2F1* (Figure 5C). Using the same parameters, the pseudotime calculation was also performed in CD4$^+$ and CD8$^+$ CAR-T cells, separately (Figure S4).

Differentially-expressed genes were identified with more than 1.25 fold change and p<0.05 with two-sided *t*-test by comparing one group with others (Figure 5F). T cell exhaustion gene signatures were obtained from the literature (Belk et al. 2022). The enrichment of T cell exhaustion gene signatures was assessed to genes sorted by Pearson correlation coefficients with the inferred antitumor score or pseudotime by GSEA (v4.1.10) (Subramanian et al. 2005). 0.05 FDR was used as a cutoff of statistical significance (Figure 5G).

Reference

Adams L, Song MK, Yuen S, Tanaka Y, Kim YS. 2024. A single-nuclei paired multiomic analysis of the human midbrain reveals age- and Parkinson's disease-associated glial changes. *Nat Aging* **4**: 364-378.

Alquicira-Hernandez J, Sathe A, Ji HP, Nguyen Q, Powell JE. 2019. scPred: accurate supervised method for cell-type classification from single-cell RNA-seq data. *Genome Biol* **20**: 264.

Belk JA, Yao W, Ly N, Freitas KA, Chen YT, Shi Q, Valencia AM, Shifrut E, Kale N, Yost KE et al. 2022. Genome-wide CRISPR screens of T cell exhaustion identify chromatin remodeling factors that limit T cell persistence. *Cancer Cell* **40**: 768-786 e767.

Bhaduri A, Di Lullo E, Jung D, Müller S, Crouch EE, Espinosa CS, Ozawa T, Alvarado B, Spatazza J, Cadwell CR et al. 2020. Outer Radial Glia-like Cancer Stem Cells Contribute to Heterogeneity of Glioblastoma. *Cell Stem Cell* **26**: 48-63.e46.

Cao J, Spielmann M, Qiu X, Huang X, Ibrahim DM, Hill AJ, Zhang F, Mundlos S, Christiansen L, Steemers FJ et al. 2019. The single-cell transcriptional landscape of mammalian organogenesis. *Nature* **566**: 496-502.

Dann E, Henderson NC, Teichmann SA, Morgan MD, Marioni JC. 2022. Differential abundance testing on single-cell data using k-nearest neighbor graphs. *Nat Biotechnol* **40**: 245-253.

Falcon S, Gentleman R. 2007. Using GOstats to test gene lists for GO term association. *Bioinformatics* **23**: 257-258.

Hafemeister C, Satija R. 2019. Normalization and variance stabilization of single-cell RNA-seq data using regularized negative binomial regression. *Genome Biol* **20**: 296.

Kelava I, Chiaradia I, Pellegrini L, Kalinka AT, Lancaster MA. 2022. Androgens increase excitatory neurogenic potential in human brain organoids. *Nature* **602**: 112-116.

Mule MP, Martins AJ, Tsang JS. 2022. Normalizing and denoising protein expression data from droplet-based single cell profiling. *Nat Commun* **13**: 2099.

Smajic S, Prada-Medina CA, Landoulsi Z, Ghelfi J, Delcambre S, Dietrich C, Jarazo J, Henck J, Balachandran S, Pachchek S et al. 2022. Single-cell sequencing of human midbrain reveals glial activation and a Parkinson-specific neuronal state. *Brain* **145**: 964-978.

Smith AM, Davey K, Tsartsalis S, Khozoie C, Fancy N, Tang SS, Liaptsi E, Weinert M, McGarry A, Muirhead RCJ et al. 2022. Diverse human astrocyte and microglial transcriptional responses to Alzheimer's pathology. *Acta Neuropathol* **143**: 75-91.

Subramanian A, Tamayo P, Mootha VK, Mukherjee S, Ebert BL, Gillette MA, Paulovich A, Pomeroy SL, Golub TR, Lander ES et al. 2005. Gene set enrichment analysis: a knowledge-based approach for interpreting genome-wide expression profiles. *Proc Natl Acad Sci U S A* **102**: 15545-15550.

Tian M, Cheuk AT, Wei JS, Abdelmaksoud A, Chou HC, Milewski D, Kelly MC, Song YK, Dower CM, Li N et al. 2022. An optimized bicistronic chimeric antigen receptor against GPC2 or CD276 overcomes heterogeneous expression in neuroblastoma. *J Clin Invest* **132**.

Velmeshev D, Schirmer L, Jung D, Haeussler M, Perez Y, Mayer S, Bhaduri A, Goyal N, Rowitch DH, Kriegstein AR. 2019. Single-cell genomics identifies cell type-specific molecular changes in autism. *Science* **364**: 685-689.

Verhaak RG, Hoadley KA, Purdom E, Wang V, Qi Y, Wilkerson MD, Miller CR, Ding L, Golub T, Mesirov JP et al. 2010. Integrated genomic analysis identifies clinically relevant subtypes of glioblastoma characterized by abnormalities in PDGFRA, IDH1, EGFR, and NF1. *Cancer Cell* **17**: 98-110.

Wang Q, Hu B, Hu X, Kim H, Squatrito M, Scarpace L, deCarvalho AC, Lyu S, Li P, Li Y et al. 2017. Tumor Evolution of Glioma-Intrinsic Gene Expression Subtypes Associates with Immunological Changes in the Microenvironment. *Cancer Cell* **32**: 42-56.e46.

Xu J, Farsad HL, Hou Y, Barclay K, Lopez BA, Yamada S, Saliu IO, Shi Y, Knight WC, Bateman RJ et al. 2023. Human striatal glia differentially contribute to AD- and PD-specific neurodegeneration. *Nat Aging* **3**: 346-365.
