# Supplementary figures and images for: Inferring disease progression stages in single-cell transcriptomics using a weakly supervised deep learning approach
Source: Genome Res. 2025 Jan;35(1):135–46. doi: 10.1101/gr.278812.123 (PMC11789631; doi:10.1101/gr.278812.123)

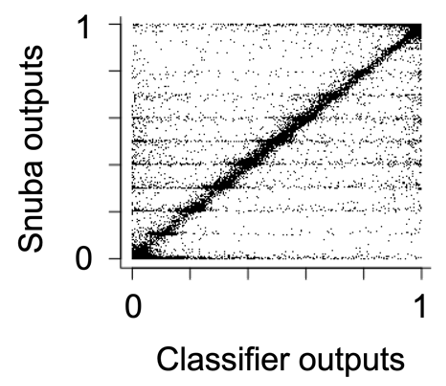

Supplement: Supplement 4 [file Supplemental_Code_S1.zip › scIDST-main/Fig/Appendix1.png]

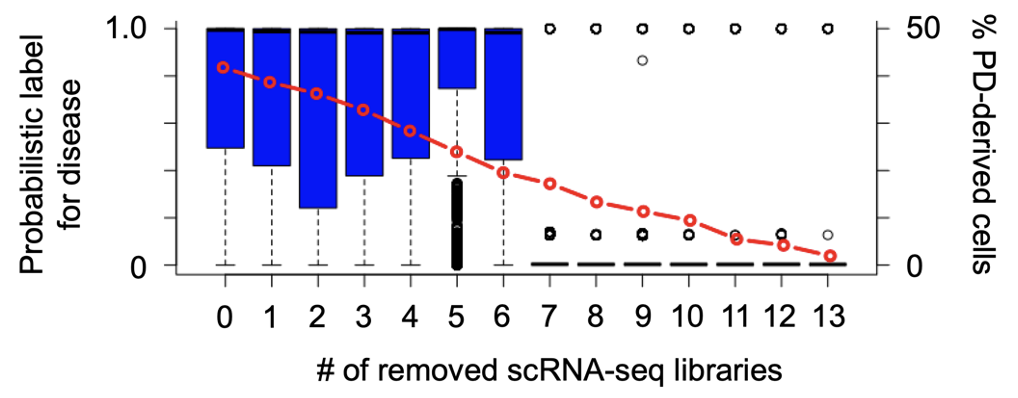

Supplement: Supplement 4 [file Supplemental_Code_S1.zip › scIDST-main/Fig/Appendix2.png]

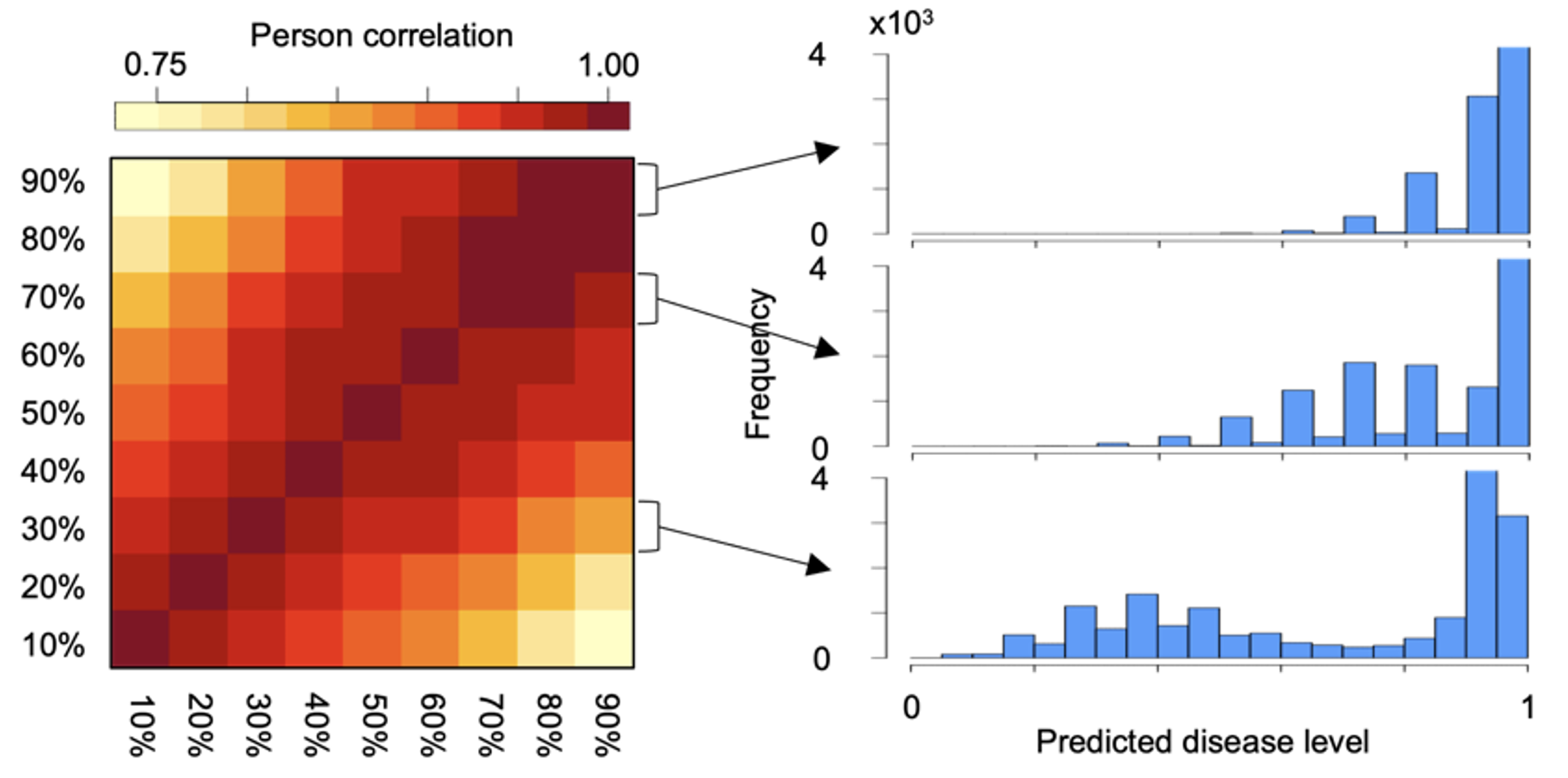

Supplement: Supplement 4 [file Supplemental_Code_S1.zip › scIDST-main/Fig/Appendix3.png]

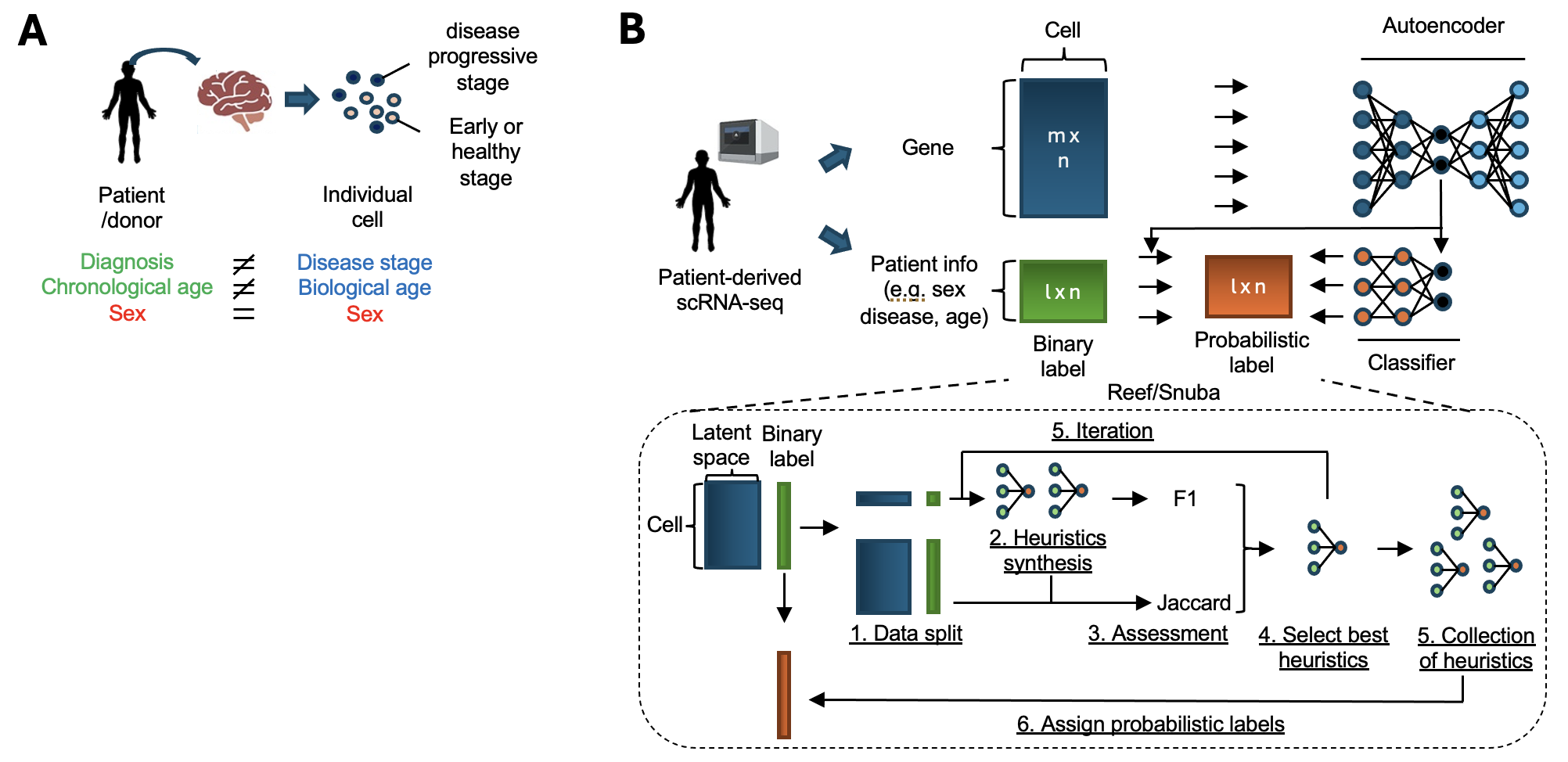

Supplement: Supplement 4 [file Supplemental_Code_S1.zip › scIDST-main/Fig/model.png]
